# Appraisals Generate Specific Configurations of Facial Muscle Movements in a Gambling Task: Evidence for the Component Process Model of Emotion

**DOI:** 10.1371/journal.pone.0135837

**Published:** 2015-08-21

**Authors:** Kornelia Gentsch, Didier Grandjean, Klaus R. Scherer

**Affiliations:** 1 Swiss Center for Affective Sciences (CISA), University of Geneva, Geneva, Switzerland; 2 Neuroscience of Emotion and Affective Dynamics Lab (NEAD), Department of Psychology, University of Geneva, Geneva, Switzerland; University of Ariel, ISRAEL

## Abstract

Scherer’s Component Process Model provides a theoretical framework for research on the production mechanism of emotion and facial emotional expression. The model predicts that appraisal results drive facial expressions, which unfold sequentially and cumulatively over time. In two experiments, we examined facial muscle activity changes (via facial electromyography recordings over the corrugator, cheek, and frontalis regions) in response to events in a gambling task. These events were experimentally manipulated feedback stimuli which presented simultaneous information directly affecting goal conduciveness (gambling outcome: win, loss, or break-even) and power appraisals (Experiment 1 and 2), as well as control appraisal (Experiment 2). We repeatedly found main effects of goal conduciveness (starting ~600 ms), and power appraisals (starting ~800 ms after feedback onset). Control appraisal main effects were inconclusive. Interaction effects of goal conduciveness and power appraisals were obtained in both experiments (Experiment 1: over the corrugator and cheek regions; Experiment 2: over the frontalis region) suggesting amplified goal conduciveness effects when power was high in contrast to invariant goal conduciveness effects when power was low. Also an interaction of goal conduciveness and control appraisals was found over the cheek region, showing differential goal conduciveness effects when control was high and invariant effects when control was low. These interaction effects suggest that the appraisal of having sufficient control or power affects facial responses towards gambling outcomes. The result pattern suggests that corrugator and frontalis regions are primarily related to cognitive operations that process motivational pertinence, whereas the cheek region would be more influenced by coping implications. Our results provide first evidence demonstrating that cognitive-evaluative mechanisms related to goal conduciveness, control, and power appraisals affect facial expressions dynamically over time, immediately after an event is perceived. In addition, our results provide further indications for the chronography of appraisal-driven facial movements and the underlying cognitive processes.

## Introduction

To understand how a person feels about an event, we often look at the face to detect changes in expression. For example, when we watch a friend’s face while s/he is answering a phone call, a frown often means bad news, whereas a smile or a relaxed face tends to indicate good news. The face provides rapid nonverbal information about a person’s emotional reaction. The study of the information revealed by facial expressions has a long history. Most of this research has focused on the recognition of emotional facial expressions, but has largely neglected investigating the underlying production mechanisms (for a recent review, see [1]). Therefore, neither the nature of the production mechanisms of facial expressions nor the type of message communicated is well understood.

Emotion theories differ in their assumptions about the function and meaning of facial expressions (for details, see [2]). For example, the basic emotions approach claims that facial expressions communicate prototypical patterns of a limited set of emotions (e.g., [3, 4]). According to this notion, joy is always expressed through smiling and simultaneous wrinkling of the corner of the eyes; sadness is characterized by lifting and knitting of the eyebrows and lowering of the mouth corners. The central notion is that each emotion is characterized by a prototypical facial expression. This link between an emotion and a particular facial expression is expected to be innate and largely independent of culture. The finding that congenitally blind children and adults can produce voluntary facial expressions of different basic emotions such as joy, anger, or sadness supports the idea of innateness [5–7]. On the other hand, empirical studies provide inconsistent evidence for the claim of cultural influences on facial expressions (for a recent review, see [8, 9]), but it seems that social contexts affect facial expressions and their recognition. However, the results indicate some degree of innateness of facial expressions, which implies that the underlying production mechanisms might be universal.

In contrast to the basic emotions approach, appraisal theories propose that facial expressions communicate cognitive elements in the emotion process. These elements are the result of a person’s *appraisal* of a given situation (e.g., [10–13]). Given that appraisals are highly subjective, depending on the individual’s needs, goals, values, and coping potential, facial expressions in emotion episodes can be expected to be distinctive to the individual and the context [2]. Appraisal theories make specific predictions about underlying production mechanisms of facial expressions, claiming that appraisal results and their efferent effects on physiological arousal and action tendencies (e.g., sensitization for visual, auditory, and olfactory stimuli) drive the innervation of facial muscles. While appraisal theorists agree on the underlying mechanism, there are divergent opinions on the nature of the appraisal process (see [10]): Contrary to other appraisal theorists who do not specify the detailed process of appraising relevant events, Scherer [12, 14, 15] proposed in his Component Process Model of emotion (CPM) that different appraisal checks (focusing on different evaluative criteria or dimensions) occur sequentially and that the nature of the emotional experience changes each time a new appraisal result modifies the overall evaluation.

According to the CPM [12, 14, 15], the first appraisal in the sequence is that of novelty—something in the environment (physical, social, or mental) occurs suddenly and unexpectedly, attracting the attention of the organism. If the eliciting stimulus cannot be disregarded as irrelevant to well-being, further appraisal checking will occur. In the next step, the intrinsic pleasantness or unpleasantness of the stimulus is evaluated, often so rapidly that it is subjectively indistinguishable from the experience of attention. Especially in the case of unpleasant stimuli, further appraisals ensue, and the emotional experience changes from feeling good or feeling bad to some more differentiated state. Subsequent appraisals check the following aspects: Is this important to me? (relevance). Do I understand what is going on? (certainty, predictability). Is something impeding my progress towards a goal or facilitating it? (goal conduciveness). What caused this to happen? (agency: self, other, or chance). Can the consequences be controlled? (control). Do I have the necessary resources to exert control (power)? Has a social norm or personal values been violated? (compatibility with standards). Different combinations of outcomes for these appraisal checks characterize different emotions.

The CPM further holds that these appraisal processes occur at different levels of cognitive information processing and includes unconscious processing (cf. [16]). Whether the appraisals always occur in the same sequence or whether sequences can be variable is a matter of debate, as is the issue of whether all of the appraisals must always occur (e.g., [10, 11]). The experimental investigation of these theoretical claims is difficult because of the rapidity of the process and the limited access to consciousness, but several electroencephalography (EEG) studies provide evidence for sequential appraisal processing at the brain level [17–19]. The organization of efferent appraisal effects is largely unexplored, except for a few studies investigating the unfolding of efferent appraisal effects in facial expressions and facial muscle activity changes [13, 20–23]. Investigating the unfolding of the innervation of specific facial muscle groups as a proxy to determine how efferent appraisal effects drive facial expression hold promise to better understand the organization between central and peripheral effects during emotional episodes (assuming that specific types of facial expression reflect underlying appraisals and consequent actions, e.g., pulling the eyebrows up to facilitate visual perception [24]). Measuring the innervation of the facial musculature with the help of facial electromyography (EMG) represents a promising methodological approach given its high temporal resolution and high sensitivity, which allows detecting subtle changes over a facial region in the absence of overt facial expressions.

### Link between Appraisals and Facial Expressions

To date, only a handful of EMG studies conducted in the appraisal framework have experimentally studied the link between specific appraisals and the resulting facial expressions [13, 20–23]. First direct evidence for the postulated link between cognitive operation of appraisals and facial muscle activity changes was reported by Smith [13]. Then, Delplanque and collaborators showed sequential effects of novelty and pleasantness appraisals in response to odors [20]. Aue, Flykt, and Scherer [21] presented emotional pictures varying in relevance (cultural vs. biological threat vs. neutral) and simultaneously manipulated goal conduciveness appraisal (win vs. loss) with symbols superimposed on the pictures. Results showed appraisal-driven effects in facial muscle activity changes that are in line with the predicted sequential unfolding (i.e., the sequence hypothesis of the CPM). Moreover, a marginally significant interaction between relevance and goal conduciveness appraisals with time was found over the corrugator region, suggesting a cumulative appraisal-driven effect that unfolds differentially over time. In a follow-up study, Aue and Scherer [22] presented pictures that varied in intrinsic pleasantness and simultaneously goal conduciveness was manipulated. They found appraisal-specific response patterns and a significant interaction between intrinsic pleasantness and goal conduciveness appraisals over the corrugator region confirming a cumulative appraisal-driven effect. Lanctôt and Hess [23] manipulated intrinsic pleasantness and goal conduciveness appraisals in a computer game providing further supporting evidence for the sequence hypothesis.

However, to date only van Reekum [25] has manipulated, in two separate unpublished studies using a computer game setting, both goal conduciveness (Studies 1 and 2) and power appraisals (Study 2). The results of both studies suggest that goal obstructive events (losing points) prompted greater corrugator activity (frowning) than goal conducive events (winning points). In Study 1, significantly larger activity was found in response to goal obstructive than to goal conducive events over the cheek region. This uncommon finding indicates that cheek region activity can be larger in response to negative events indeed, which is in line with a reported curvilinear valence effect (i.e., higher cheek region activity following both positive and negative events, cf. [26]). However, this effect was not replicated in Study 2. Effects of power appraisal were inconclusive over the corrugator and cheek regions. Most likely, the statistical power (12 repeated trials each for low and high power) was too low to allow detecting significant differences in facial reactions related to power appraisal.

Thus, the inconclusive effects of power appraisal on facial EMG in the single unpublished study to date do not allow drawing any conclusions as to whether the cognitive processes underlying power appraisal are likely to be expressed in the face. In addition to potential effects of power appraisal on the corrugator and cheek regions, one would expect effects on other facial regions such as the frontalis region [12, 27]. So far, facial EMG has not been used to examine possible effects of power appraisal over the frontalis region although the CPM predicts that low power appraisal should drive eyebrow raising (see [12, 27]).

Taken together, the results of these studies suggest two major findings: (1) Appraisal results of novelty, intrinsic pleasantness, relevance, and goal conduciveness/obstructiveness can be shown to drive specific facial expressions; and (2) these effects unfold sequentially over time in a fixed order, as predicted by the CPM. Furthermore, the order of the sequential effects in the unfolding of facial muscle activity changes seems to be largely independent of specific manipulations of experimental tasks (e.g., different kinds of operationalization for studying goal conduciveness) and of sensory domain (vision, olfaction, audition, or imagination) in which the appraisals were manipulated, suggesting a high degree of generalizability of the findings. The results reported in the cited studies above, suggest the following appraisal onset times for specific facial expressions: novelty (~100 ms eyebrow raising for novel events), relevance (~800 ms highest muscle tone relaxation for irrelevant events), pleasantness (~400–700 ms frowning for unpleasant events, ~2 s smiling for pleasant events), and goal conduciveness (~800–1000 ms frowning for goal obstructive events; ~800–1000 ms smiling for goal conducive events).

Summarizing the results of these earlier EMG studies, the following conclusions can be drawn for the facial regions investigated so far: Over the *corrugator region*, appraisal effects of relevance (larger activity following relevant than irrelevant events, ~400 ms [21]), intrinsic pleasantness (larger activity following unpleasant than pleasant events, ~400 ms [20, 22, 23]), and goal conduciveness appraisal (larger activity following goal obstructive than goal conducive events, ~800 ms [22, 23]) were found. Based on the empirical evidence it is possible that these effects reflect a one-to-one mapping of each appraisal criterion (cf. [21]), since predominantly main effects were found. Interestingly, one study reported a significant cumulative interaction effect of intrinsic pleasantness and goal conduciveness appraisals [22]. The presence of this interaction effect suggests that the effects over the corrugator region might consist of a *final integrative pattern* reflecting the nature of the event based on the cumulative effect of several appraisal criteria (cf. [21]). To date, no systematic investigation of interaction effects of two or three appraisal criteria in facial EMG recordings has been performed. It is an open question whether appraisal criteria combine in an additive, multiplicative, or other manner, and whether those cumulative effects differ among facial regions.

Over the *cheek region*, appraisal effects were mixed. In most of the reviewed studies [21–23] larger activity was found following goal conducive than goal obstructive events (~800 ms). However, in a few studies the pattern was reversed (e.g., [25, 26]), suggesting that the cheek region shows a curvilinear response pattern. Also intrinsic pleasantness appraisal affected cheek region muscle activity in the form of larger activity following pleasant than unpleasant events, ~400 ms [22, 23].

Concerning the *frontalis region*, since Darwin [28], eyebrow raising is often associated with a facial expression of surprise (implying a novelty or unpredictability appraisal). However, the signaling function of the eyebrow raise has been controversial in emotion research, the empirical evidence being quite inconclusive [29–31]. However, the work of Delplanque and collaborators [20] using novelty appraisal manipulation and facial EMG recordings clearly demonstrated novelty appraisal effects over the frontalis region. The CPM predicts (for details, see [27]) that eyebrow raising should be linked to low power appraisals when events are in principle controllable because the individual has insufficient power to ward off danger in those events. Consequently, protective responses are indicated with flight or subordination as expected action tendencies and with resulting facial expressions of submission or subordination, characterized among others by brow raising.

In conclusion, the experimental work reported here showed reliable sequential effects of different appraisal criteria. Effects of power appraisal on facial expressions over the corrugator and cheek regions using EMG have been studied only once, resulting in inconclusive findings. Moreover, the question concerning the onset time of power appraisal effects remain to be investigated as well as their potential effect on other facial regions such as the frontalis region. Given the state of the literature further empirical investigations are needed.

### Production Mechanism of Facial Expressions

Most of the studies described above empirically tested theoretical predictions of the CPM [12, 14, 27] and the appraisal model by Smith and Ellsworth [32]. Like other appraisal theories (e.g., [32–36]), the CPM considers appraisal to be the predominant cause of emotion elicitation and differentiation, driving facial expressions. Importantly, the model also holds—in contrast to other theories which do not address the underlying mechanism of facial expressions—that appraisal results drive facial expression sequentially and cumulatively as soon as processing of an appraisal criterion reaches preliminary closure (for details, see[12]). Preliminary closure refers to a sufficiently conclusive appraisal result to warrant efferent (appraisal-driven) commands to be sent to facial muscle regions [12, 14, 15]. Clear experimental support for this prediction has been found in several studies, for example, a conclusive appraisal result of goal obstruction triggers a frown [13, 21, 23].

**Fig 1:**
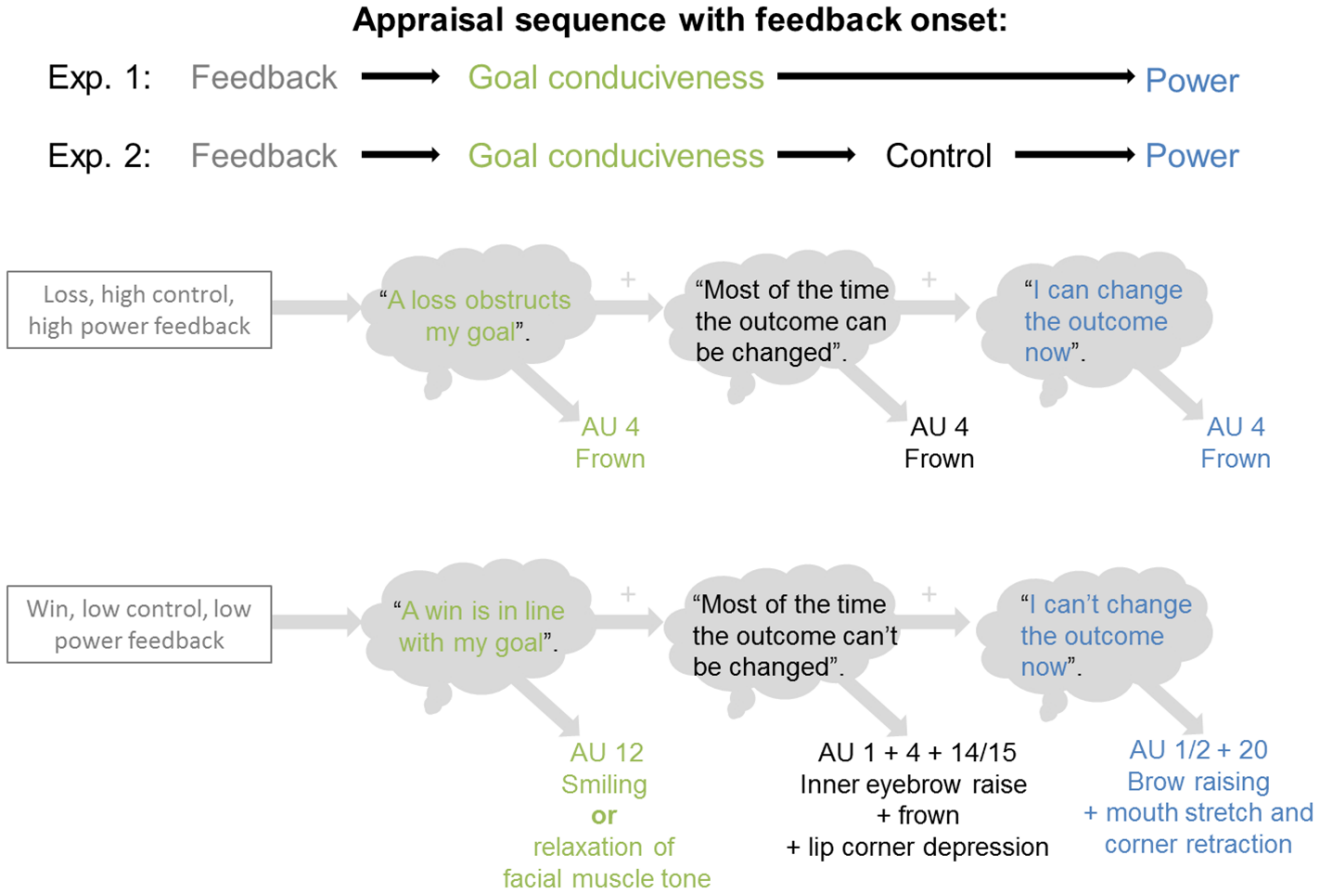
Illustration of sequential and cumulative effects of appraisals on facial expressions predicted by the Component Process Model. The numbers indicate the Action Unit (AU) according to the Faction Action Coding System [37] which are accompanied by descriptions of the respective facial expression.

In contrast to other appraisal theories, the CPM provides detailed predictions about which specific appraisal results drive the innervation of specific facial muscle groups. Therefore, it has been the model chosen to inform most of the studies reported in this article. The model specifies appraisal-driven effects on facial regions (see component patterning predictions, most recent version in [12]) which can be directly empirically tested. No predictions have been made on the exact timing of appraisal on facial expressions due to the lack of pertinent work in the literature. According to the unique sequence hypothesis proposed by the model, facial expressions unfold over time in a particular fixed sequence (see Fig 1): Each appraisal result differentially and cumulatively affects facial expressions.

The sequence hypothesis of the CPM is based on logical implications of how cognitive inferential processes are hierarchically structured, as well as on the phylogenetic and ontogenetic progression of emotion expression (for more details, see [14, 15, 27]). Logical implications refer to the necessity of sequentially processed information to achieve conclusive inferences about events. For example, to determine the response options for an event (i.e., control and power appraisals), it is crucial to know whether the event is relevant and whether it helps or hinders one from reaching a current goal. In particular, when goal attainment is blocked, further actions to change the event or its consequences become important. Consequently, to determine the response options (control and power appraisals), the preceding preliminary closure of appraisals including relevance and goal conduciveness must be available. Regarding the ontogenetic development of facial expressions in infants, the model states that neural structures related to appraisal processes develop in stages [12, 14]. For example, neural structures involved in novelty appraisal are predicted to develop earlier in life than those of goal conduciveness or control and power appraisals. As a result, it is predicted that infants express surprise (novelty appraisal) at an earlier age than anger (appraisals of goal obstruction, high control, and high power) [38].

### Control and Power Appraisals

Control and power appraisals are of central importance for the study of emotion and related facial expressions. Nonetheless, they are rather less frequently investigated compared to intrinsic pleasantness and goal conduciveness appraisals. Appraisal theories [15, 33, 34] predict that the broad categorization into positive and negative emotions and their facial expressions principally depends on the appraisals of intrinsic pleasantness and goal conduciveness (or motivational incongruence, cf. [13, 39]). However, the differentiation of the different types of positive and particularly of negative emotions depends on control (i.e., the degree to which outcomes are perceived to be controllable by human action in the given situation) and power appraisals (i.e., personal ability appraisal with respect to the resources at one’s disposal to change contingencies and outcomes in line with current goals) [14, 15, 27, 33, 34]. For example, control and power appraisals can be conceptualized as follows for different negative emotions such as sadness (low control and low power), anger (high control and high power), or fear (high control and low power).

Another important reason to study control and power appraisals results from the vantage point of the sequence hypothesis of the CPM: the need to determine the unfolding of these appraisal-driven effects on facial expressions. While the sequence hypothesis has been repeatedly investigated for novelty, relevance, intrinsic pleasantness, and goal conduciveness appraisals, a comprehensive investigation of the subsequent appraisals of the sequence—in particular of control and power appraisals—is lacking.

### The Present Study

To extend empirical testing of predictions concerning the appraisal-based production mechanisms of facial expressions, we conducted two experiments. In these experiments, goal conduciveness (Experiments 1 and 2), power (Experiments 1 and 2), and control (Experiment 2) appraisals were manipulated. The experiments were designed to test two predictions of the CPM: (1) that the results of power and control appraisals directly drive facial expressions and (2) that these appraisal effects occur subsequent to goal conduciveness effects.

Goal conduciveness, control, and power appraisals were manipulated via systematically varied outcome feedback in a gambling task. Feedback-locked facial EMG recordings over the frontalis, corrugator, and cheek regions were analyzed. According to the CPM (see [12]), goal conduciveness appraisal evaluates whether an event is conducive or obstructive for attaining current goals. Gambling outcomes presented to participants manipulated goal conduciveness appraisal: Wins were expected to be goal conducive, losses to be goal obstructive and break-even considered an intermediate condition (for similar manipulations in an EEG study, see [17]). The CPM conceptualizes two appraisals for determining the response options (i.e., coping potential): control appraisal (the degree to which outcomes are perceived to be controllable by human action in the given situation) and power appraisal (personal ability appraisal with respect to the resources at one’s disposal to change contingencies and outcomes according to current goals). In the gambling task, power appraisal was operationalized as being given the choice to freely decide about the gambling outcome at the end of each trial. Participants had high power when they could freely choose the gambling outcome; in contrast, they had low power when they were unable to choose it. Control appraisal was manipulated across trials within blocks. Thus, participants perceived high or low situational control, depending on the respective gambling block.

The EMG recording sites were selected on the basis of the componential patterning assumptions of the CPM (see Table 1 in [40]). Below, Table 1 presents global predictions of the effects of goal conduciveness, control, and power appraisals on facial expressions. No predictions are formulated with respect to cumulative appraisal effects. For goal conduciveness appraisal, we predicted the following effects (see also Table 1, below): Goal conducive events (wins) are expected to relax the facial muscle tone over the eyebrow region (resulting in decreased corrugator activity, around or below zero, ~600 ms) and to generate smiling (increased muscle activity over the cheek region, ~600–800 ms). In contrast, goal obstructive events (losses) are expected to produce frowning (increased corrugator activity, ~600–800 ms). No theoretical predictions were made for break-even outcomes.

**Table 1:** Component Patterning Theory Predictions for Facial Action Units (AUs) Following Appraisal Outcomes.

Appraisal criterion	Predicted facial expression in terms of Action Units (and expressive behavior)
Goal conduciveness	
Conducive	AUs 5 (lids up), 26 (jaw drop, open mouth), 38 (open nostrils); or **12 (lip corners pulled upward)**, 25 (lips part)
Obstructive	**AUs 4 (frown)**, 7 (lids tighten), 23 (lips tighten), 17 (chin raising)
No Control	Hypotonus of facial musculature, AUs **15 (lip comer depression)**, 25 (lips parting), 26 (jaw dropping), 41 (lids drooping), 43 (eyes closed); or **1 (inner brow raise)**, **4 (brow lowered)**
Control is possible and	
High power	**AUs 4**, 5 (**eyebrows contracted**, eyes widened); or 7 (lids tightened, eyes narrowed), 23, 25 (lips tight and parted, bared teeth); or 23, 24 (lips tight, pressed together), 38 (nostril dilation)
Low power	**AUs 1 + 2 +** 5 (**brow** and lid **raising**), 26 (jaw drop), **20 (mouth stretch and corner retraction)**, 38 (nostril dilation)

The relevant predictions for Experiments 1 and 2 are highlighted in bold characters. The EMG recordings over the frontalis, corrugator, and cheek regions in the present experiments are an approximate measure of the Actions Units that are in bold characters.

For power and control appraisals, we predicted the following response patterns for specific facial regions (see also Table 1): (a) Appraisal results of low power or low control are expected to trigger raising of the eyebrows (resulting in increased frontalis activity, earliest at ~800 ms); (b) high power appraisal is expected to elicit frowning (resulting in increased corrugator activity, earliest at ~800 ms), whereas high and low control appraisals should lead to contracted and lowered eyebrows, respectively (both resulting in increased corrugator activity, earliest at ~800 ms); and (c) low power appraisal is expected to trigger mouth stretch and lip corner retraction, whereas low control appraisals cause lip corner depression (both low power and low control results were expected to increase muscle activity over the cheek region, earliest at ~800 ms).

We made the following temporal predictions of sequential appraisal effects in facial muscle activity changes. Effects of goal conduciveness appraisal should be detected at about 600–800 ms after feedback onset. Effects of control and power appraisals should be observed subsequently, about 800 ms after feedback onset at the earliest. We made no a priori predictions about interaction effects. Nonetheless, judging from the results of previous work (e.g., [22]), they would be expected to occur rather over the corrugator region than over the frontalis or cheek regions.

## Experiment 1

### Materials and Methods

#### Participants

Twenty-four female undergraduates of the University of Geneva were recruited. They were right-handed (mean of Edinburgh Handedness Inventory = 81.26, *SD* = 15.52), healthy, had normal or corrected-to-normal vision, and ranged in age from 18 to 28 years (*M* = 21.42, *SD* = 2.13). Participants were paid 30 CHF plus the bonus money they won in the task (max. 16.75 CHF). The study was fully approved by the Ethics Committee of the University of Geneva.

#### Experimental Paradigm and Procedure

**Fig 2:**
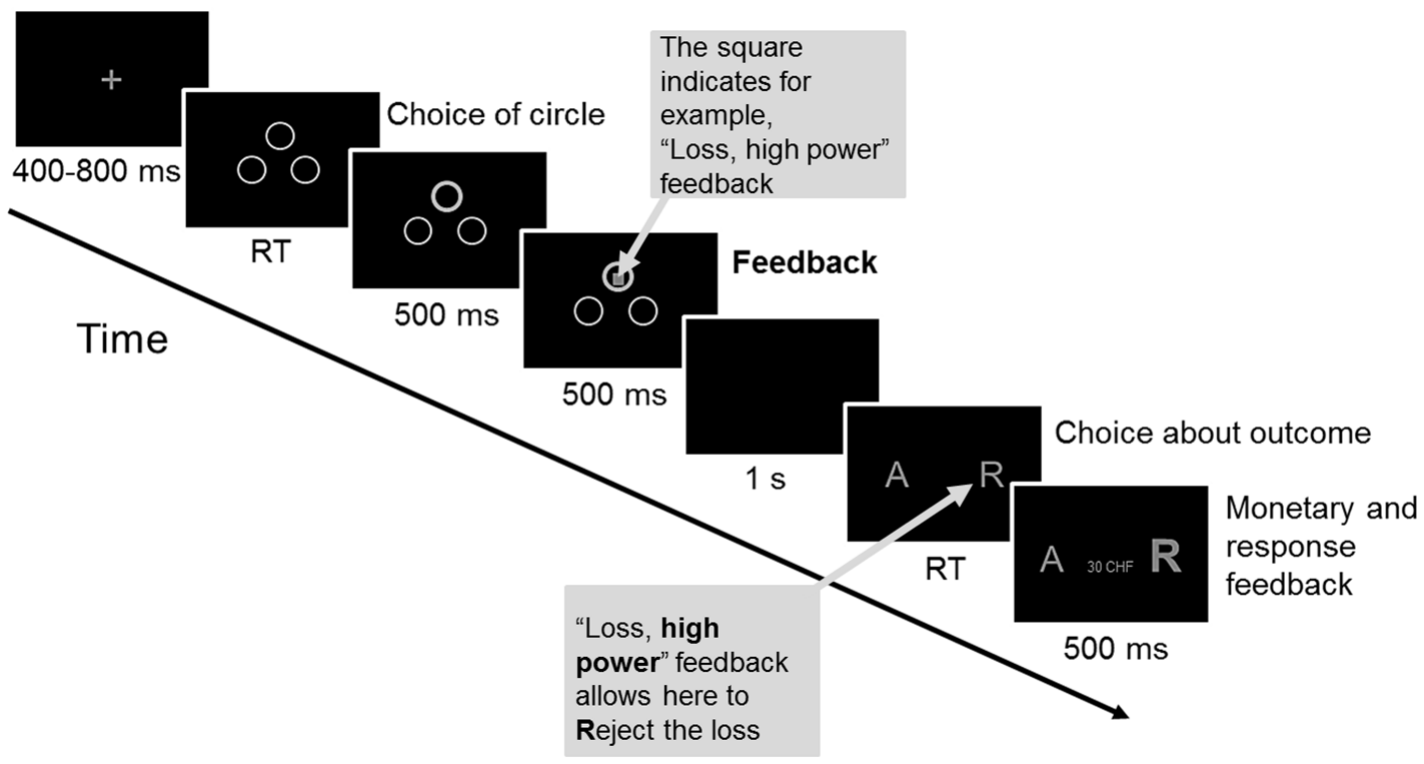
An example of a gambling task trial of Experiment 1. Presentation times of each trial event are indicated below the corresponding screen. At feedback onset, the information of goal conduciveness and power appraisals was simultaneously presented via a geometric shape in gray or black. At “Choice about outcome” participants decided about the outcome: A = accepting. R = rejecting. RT = reaction time.

After arrival at the laboratory, participants read and signed an informed consent form. They filled out questionnaires about their current health and demographic characteristics. The practice session and the experimental task took place in a sound-attenuated room. Both tasks were presented on a computer screen (17″, resolution 1280 × 1024) and contained only gray characters against a black background (Fig 2). The distance between participants’ eyes and the computer screen was 60 cm. Participants used the number pad of a standard PC keyboard to give their choices for each gambling trial.

The gambling task consisted of the following course of events within one trial, shown in Fig 2. Each trial started with a fixation cross (randomized duration 400–800 ms; 1° high, 1° wide) in the center of the screen, followed by three circles (Fig 2, screen “Choice of circle”; 3.8° high, 4.6° wide). Participants were told that the possible outcomes of a trial (win, +0.25 CHF; loss, −0.25 CHF; and break-even, 0 CHF) were concealed under these three circles; no cues were provided that allowed the participants to tell where the win was hidden. The circle that the participant had chosen was highlighted (500 ms) before the feedback stimulus appeared at its center (Fig 2, screen “Feedback”; 500 ms).

**Fig 3:**
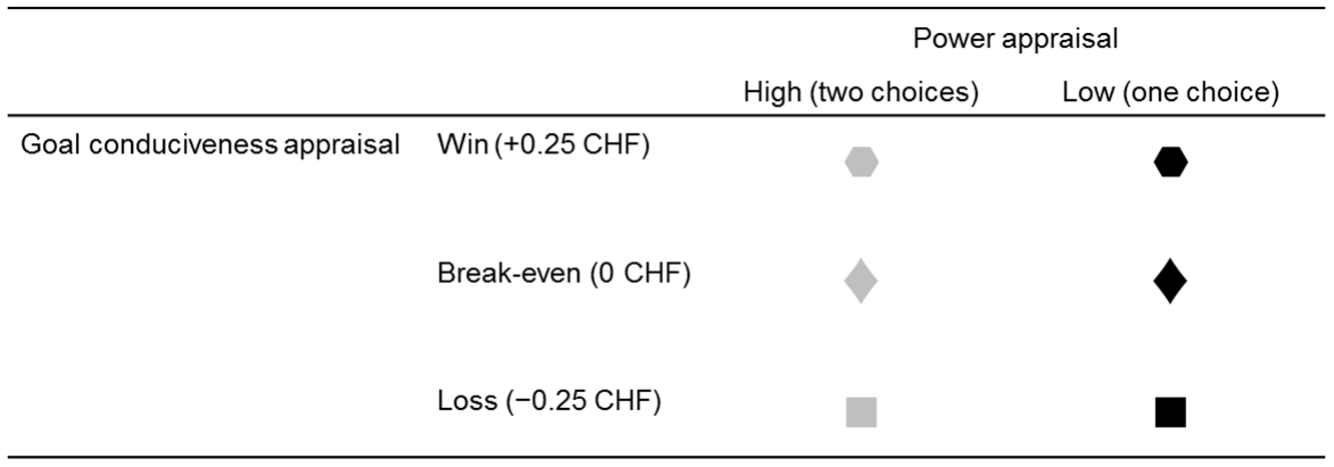
Example of feedback stimuli for the operationalization of goal conduciveness and power appraisals. The associations of the geometric shapes with the levels of goal conduciveness, and the meaning of the gray or black color of these shapes with the levels of power appraisals were counterbalanced across participants.

Feedback stimuli simultaneously conveyed information about goal conduciveness (outcome: win [goal conducive event], loss [goal obstructive event], and break-even outcome [intermediate condition]) and power (choice about the outcome at the end of a trial: choice [high power] and no choice [low power]). Consequently, the six feedback-stimuli conditions were (1) *win*, *high power*; (2) *loss*, *high power*; (3) *break-even*, *high power*; (4) *win*, *low power*; (5) *loss*, *low power*; and (6) *break-even*, *low power* (each condition was presented 64 times). Feedback stimuli were geometric shapes (hexagon, square, and diamond) with either gray- or black-colored fill. The geometric shapes (see Fig 3 as an example) encoded the three levels of goal conduciveness, whereas the color encoded the two levels of power. Feedback-stimulus probability was balanced for all feedback conditions, with equal probability and without replacement across trials.

After the feedback-stimulus presentation, the screen turned black (1 s). Next, participants were presented with the choice options for that trial. At this point, the screen had one letter to the left side and one to the right (Fig 2, screen “Choice about outcome”; A = accept, R = reject; 0.8° high, 6.6° wide; Arial font, size 28). In high power trials, participants could freely choose between two options: accepting (A) or rejecting (R) the outcome (presentation of “A R” or “R A”: randomized order with the same number of presentations). In low power trials, they had to accept the assigned option of either rejecting (presentation of “R R”) or accepting (“A A”) the outcome (randomized selection with the same number of presentations). The corresponding letter was then highlighted to accentuate the participant’s decision (Fig 2, screen “Monetary and response feedback”; 500 ms; Arial font, size 52 bold). Simultaneously, the monetary outcome of that trial was shown between these two letters. At the end of a trial, the total monetary outcome was updated to the amount of money won (+0.25 CHF) or lost (−0.25 CHF), or remained unchanged for break-even outcomes. Immediately afterward, the next trial started. In total, the gambling task (~30 min) consisted of 384 gambling trials (divided into 12 blocks of 32 trials, randomized presentation).

Prior to the gambling task, participants completed a practice session (48 trials, 5–7 min) in order to understand the rules and the meaning of the gray- and black-colored geometric shapes. To ensure that participants responded correctly, a performance cutoff criterion for high power trials was implemented (>80% of correct responses, i.e., accepting wins and rejecting losses). If the criterion was exceeded at the end of the practice session, the gambling task started after a short break; otherwise, a second practice session was run.

The amount of bonus money won during the experiment depended on the participant’s performance. Participants were instructed to win as much money as they could; the maximum bonus amount possible was not mentioned. They were assured that they would not end up losing money or owing money to the experimenter. They were not informed that the type of feedback was selected at random on each trial; they were told only that they would play a gambling game. At the end of the experiment, participants were informed about the experimental manipulations and were paid their participation fee plus the bonus money.

#### Data acquisition

The practice session and the gambling task were presented by using E-Prime 2.0 (Psychology Software Tools, Inc., Pittsburgh, PA). Facial EMG (placement of surface electrodes over frontalis, corrugator, and cheek regions according to the guidelines, [41]) were recorded and digitized (bandwidth 0.1 to 417 Hz, sampling rate: 2048 Hz) with a BioSemi Active-Two amplifier system (BioSemi Biomedical Instrumentation, Amsterdam, the Netherlands).

#### Data analysis

Preprocessing of the EMG data (using Brain Vision Analyzer software, Brain Products, Gilching, Germany) followed the standard procedure [41]. First, bipolar montages were calculated for each electrode pair of each facial region (frontalis, corrugator, and cheek region) by subtracting the recorded activity of one electrode with the activity of the neighboring electrode. Next, the continuous waveforms of the EMG data were bandpass filtered (20–400 Hz, 12 db/octave), full-wave rectified, low-pass filtered (40 Hz, 12 db/octave), and cut into segments for each experimental condition (including 500 ms baseline and 1.4 s post-stimulus intervals). The EMG data were then downsampled to 512 Hz and exported to a commercial software package (MATLAB R2012a, The MathWorks Inc., Natick, MA, 2012). Artifacts and outliers (deviating more than 2 SDs from the mean baseline activation of a given participant) were eliminated separately for each facial region (2.24% in total were removed). To examine the temporal profiles of the experimental conditions over time, we calculated mean amplitude values for the subsequent 100-ms time intervals within 1.4 s as a percentage change of the mean amplitude value relative to the baseline.

#### Statistical analyses

The EMG data of a facial region were submitted to a 3 (Goal conduciveness: win vs. loss vs. break-even) × 2 (Power: high vs. low) × 9 (Time: 100-ms time-intervals from 600 to 1400 ms based on the predictions about the timing of the effects) repeated measures multivariate analysis of variance (MANOVA). Specifically, goal conduciveness and power were treated as within-subject variables and time was introduced as a multiple dependent variable into the repeated measures MANOVA (cf. [20]). The significance of the differences among the experimental conditions was tested for each of the nine 100-ms time intervals using univariate tests (planned comparisons). Greenhouse-Geisser correction was applied whenever the assumption of sphericity was violated. The uncorrected degrees of freedom, the corrected *p* values, and the epsilon values (ε) are reported in the Results. All reported effect sizes are partial ŋ². All tests were performed at an alpha level of 5% and were computed by using IBM SPSS Statistics 22.

### Results

The results of the planned comparisons are presented in Table 2. The means and standard deviations are reported in the supporting information section (S1 Table). The data of the corrugator (S1 Data), frontalis (S2 Data), and zygomaticus (S3 Data) muscle activity changes are available in the supporting information section.

**Table 2:** Results of the Planned Comparisons for Each Facial Region.

		Corrugator region			Frontalis region			Cheek region		
	Time interval (ms)	*F*	ŋ²	ε	*F*	ŋ²	ε	*F*	ŋ²	ε
**GC**	600	5.19**	.18	.86	3.04†	.12	.86	0.49	.02	.96
	700	2.46†	.10	.87	0.13	.01	.88	1.82	.07	.83
	800	4.76*	.17	.68	0.76	.03	.88	2.63	.10	.67
	900	2.91†	.11	.90	0.22	.01	.96	2.06	.08	.70
	1000	5.61*	.20	.80	1.40	.06	.99	1.64	.07	.77
	1100	7.44**	.24	.90	0.28	.01	.91	2.60	.10	.68
	1200	5.20**	.18	.95	0.48	.02	.99	2.34	.09	.72
	1300	7.26**	.24	.91	0.59	.03	.99	0.95	.04	.72
	1400	3.46*	.13	.96	0.92	.04	.97	1.03	.04	.74
**Power**	600	1.73	.07	1.00	2.54	.10	1.00	0.06	.00	1.00
	700	1.05	.04	1.00	1.29	.05	1.00	0.15	.01	1.00
	800	5.96*	.21	1.00	3.90†	.15	1.00	0.38	.02	1.00
	900	3.29†	.13	1.00	0.48	.02	1.00	0.94	.04	1.00
	1000	0.03	.00	1.00	3.28†	.12	1.00	2.35	.09	1.00
	1100	0.07	.00	1.00	0.11	.00	1.00	2.75	.11	1.00
	1200	1.73	.07	1.00	0.06	.00	1.00	1.08	.05	1.00
	1300	4.69*	.17	1.00	1.08	.05	1.00	0.17	.01	1.00
	1400	2.14	.09	1.00	0.01	.00	1.00	0.99	.04	1.00
**GC × Power**	600	1.32	.05	.86	0.16	.01	.81	3.52*	.13	.94
	700	1.73	.07	.87	1.61	.07	.76	3.62*	.14	.86
	800	4.39*	.16	.68	1.59	.06	.88	3.72*	.14	.92
	900	3.62*	.14	.90	0.18	.01	.70	2.66†	.10	.88
	1000	0.03	.00	.80	0.73	.03	.82	2.03	.08	.72
	1100	0.04	.00	.90	1.07	.04	.91	2.50	.10	.64
	1200	0.50	.02	.95	0.56	.02	.92	3.15†	.12	.74
	1300	1.34	.06	.91	0.08	.00	.93	2.68†	.10	.75
	1400	0.37	.02	.96	1.02	.04	.86	2.49†	.10	.83

*N* = 24 (Experiment 1). For each muscle region, a repeated measures MANOVA with planned comparisons was performed. The within-subject factors were Goal conduciveness (GC: win vs. loss vs. break-even) and Power (high vs. low). Time was treated as a multiple dependent variable (nine 100-ms post-stimulus time intervals).

^†^ *p* < .10,

*p < .05,

**p < .01.

#### Goal conduciveness effects

**Fig 4:**
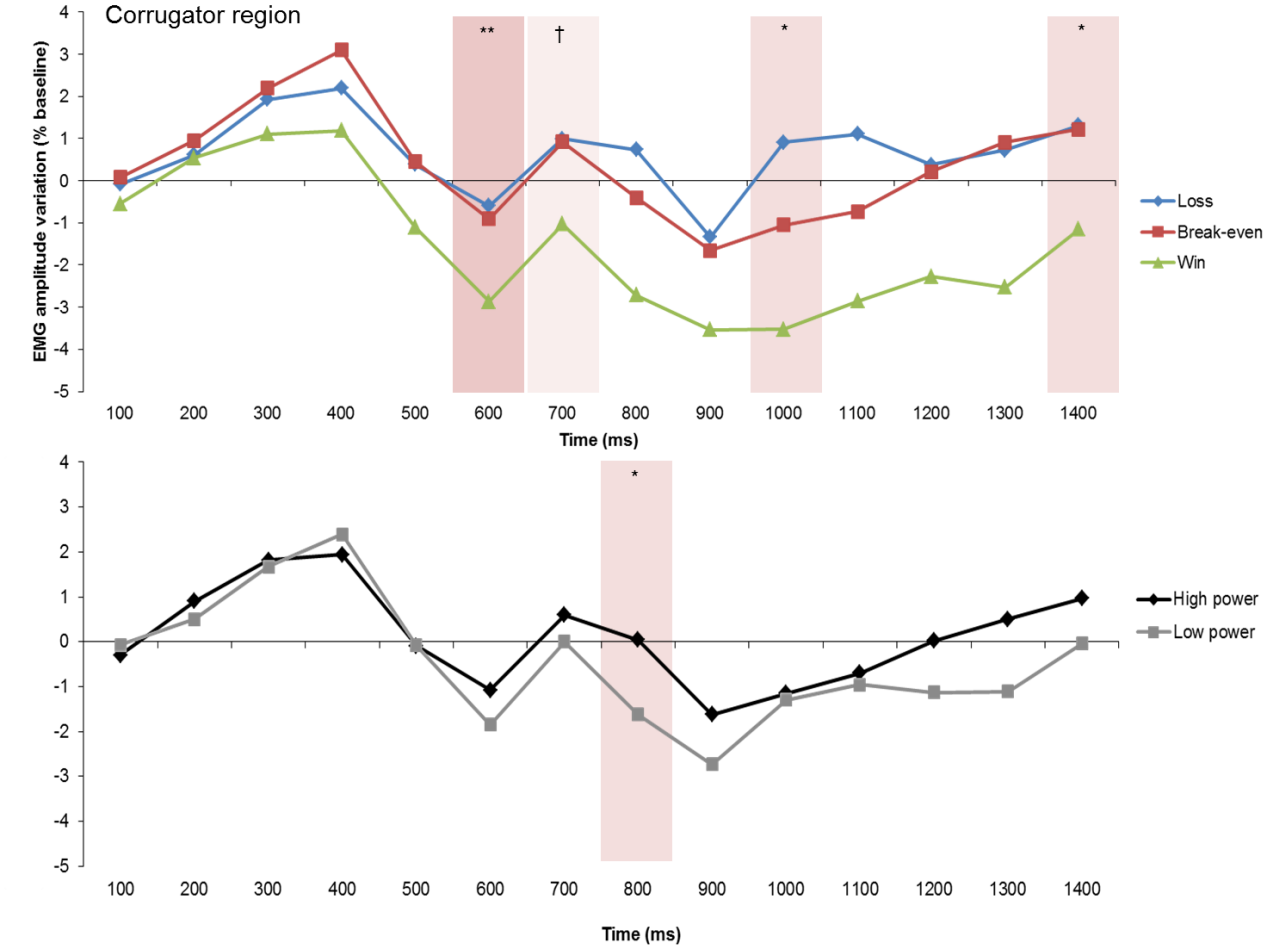
Electromyographic (EMG) amplitude variations (expressed as % change scores relative to baseline) over the corrugator region, showing the goal conduciveness and the power effects across time. †*p* < .10, *p < .05, **p < .01.

Over the corrugator region, the planned contrasts for the nine consecutive 100-ms time intervals revealed a significant main effect of goal conduciveness at 600 ms (see Fig 4, Table 2). Significant main effects were also found at 800 and 1000–1400 ms. A marginally significant main effect was observed at 700 and 900 ms. The analysis did not reveal significant results over the frontalis and cheek regions.

Pairwise comparisons of the goal conduciveness main effect over the corrugator region revealed higher corrugator activity in response to losses relative to wins at 600, 800, 1000–1400 ms, *p*s = .019, .023, .006, .001, .008, .009, and .039, respectively. Corrugator activity was also higher in response to break-even outcomes compared with wins at 600, 800–1400 ms, *p*s = .005, .003, .029, .015, .020, .007, .001, and .020, respectively. Muscle activity was similar in response to losses and break-even outcomes in all time intervals, *p*s > .124.

#### Power effects

Significant power main effects were found over the corrugator region (see Fig 4, Table 2) at 800 and 1300 ms. At 900 ms, the power effect was in tendency significant. Over the frontalis region, marginally significant power effects were obtained at 800 and 1000 ms. Over the corrugator and frontalis regions, high power elicited greater activity than lower power. Over the cheek region no significant power main effect was found.

#### Interaction effects

**Fig 5:**
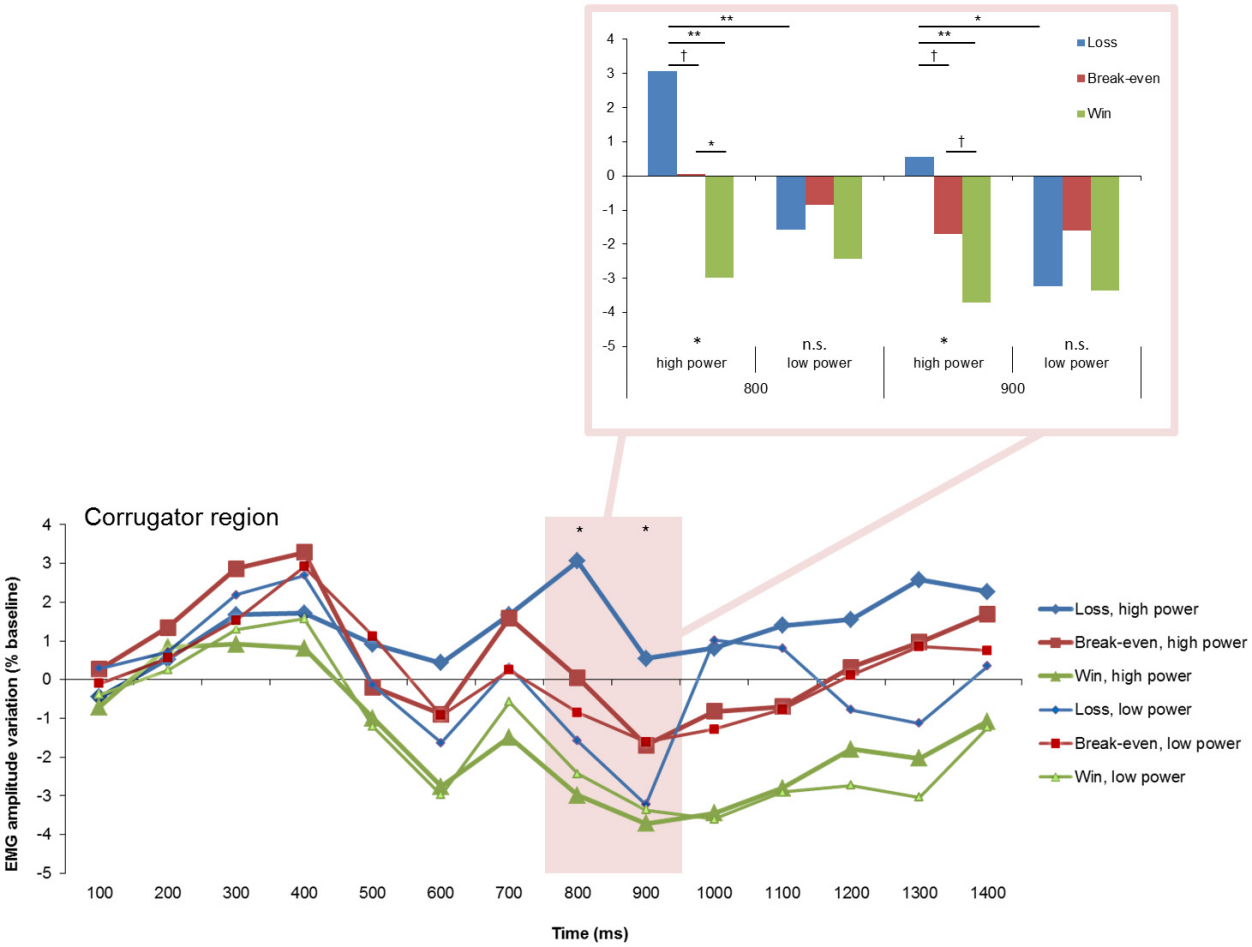
Electromyographic (EMG) amplitude variations over the corrugator region, illustrating the interaction of goal conduciveness and power appraisals across time. †*p* < .10, *p < .05, **p < .01.

**Fig 6:**
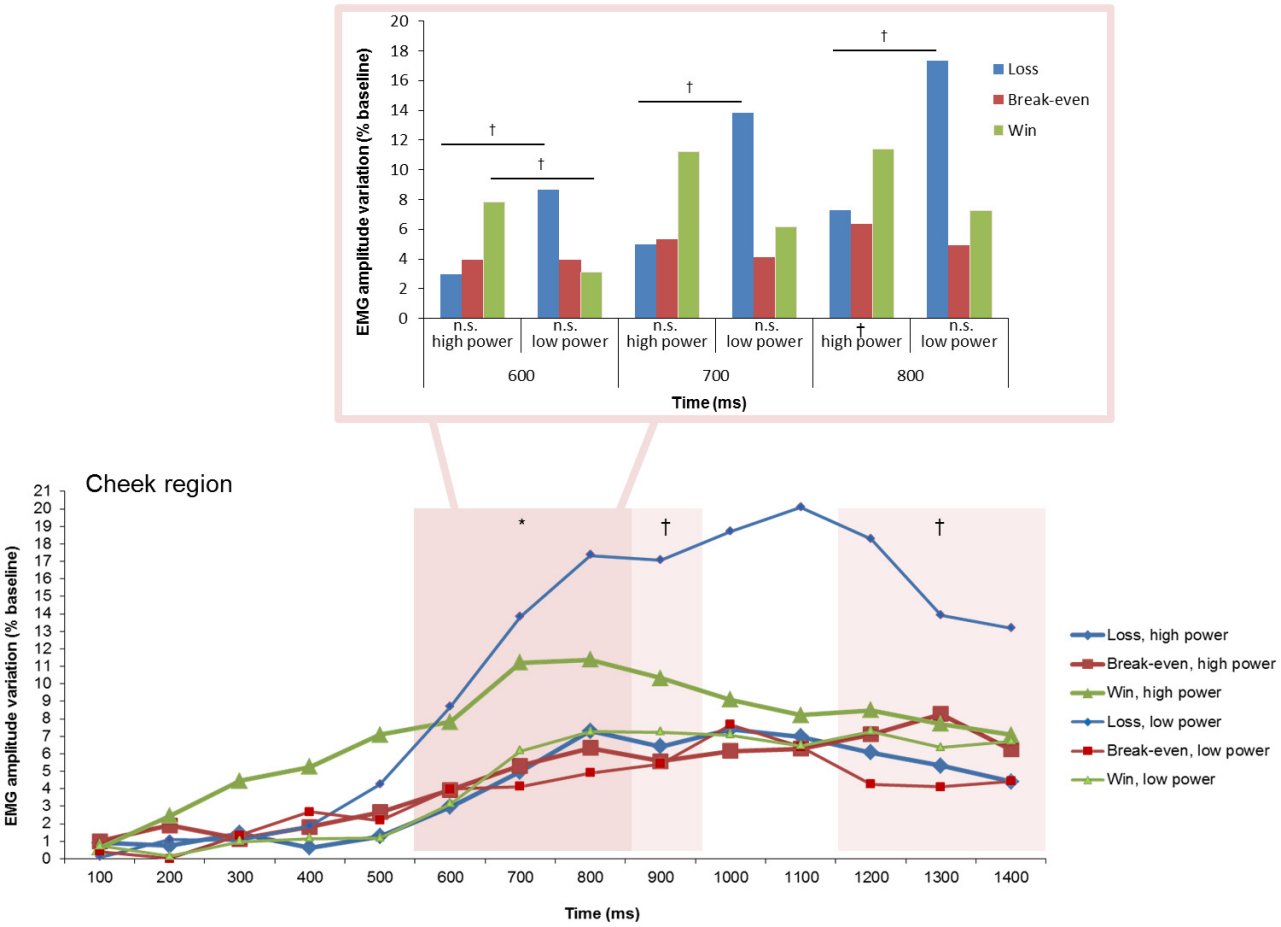
Electromyographic (EMG) amplitude variations over the cheek region, illustrating the interaction of goal conduciveness and power appraisals across time. †*p* < .10, *p < .05.

Significant interaction effects between goal conduciveness and power were found over the corrugator and cheek regions (Table 2). Over the corrugator region (Fig 5), significant effects were obtained at 800 and 900 ms. Over the cheek region (Fig 6), significant effects emerged at 600–800. At 900 and 1200–1400 ms, the interaction effects were marginally significant.

Over the corrugator region, post hoc tests investigated the interaction effect between goal conduciveness and power at 800 and 900 ms (Fig 6). At 800 ms, significant goal conduciveness effects were found for high power, *F*(2, 22) = 5.22, *p* = .014, ŋ² = .322, but not for low power (*p* = .201). Pairwise comparisons revealed that *loss*, *high power* elicited greater activity changes compared to *win*, *high power* (*p* = .007). But *loss*, *high power* had in tendency larger corrugator activity than *break-even*, *high power* (*p* = .095). *Break-even*, *high power* elicited greater activity changes than *win*, *high power* (*p* = .010). Furthermore, pairwise comparisons revealed that *loss*, *high power* elicited greater activity changes than *loss*, *low power* (*p* = .008). Win and break-even had each similar muscle activity changes in high and low power trials (*p*s = .599 and .389, respectively). At 900 ms, significant goal conduciveness effects were obtained for high power, *F*(2, 22) = 4.05, *p* = .032, ŋ² = .269, but not for low power (*p* = .351). Pairwise comparisons revealed that *loss*, *high power* elicited greater activity changes compared to *wins*, *high power* (*p* = .008). *Loss*, *high power* had again marginally greater activity changes than *break-even*, *high power* (*p* = .070). *Break-even*, *high power* elicited marginally greater activity changes than *win*, *high power* (*p* = .066). Moreover, *loss*, *high power* was associated with greater activity relative to *loss*, *low power* (*p* = .016). Muscle activity was again similar for wins and break-even in high and low power trials (*p*s = .747 and .925, respectively).

Over the cheek region, post hoc tests explored the interaction effect between goal conduciveness and power at 600, 700, and 800 ms. At 600 and 700 ms, no significant goal conduciveness effects were found for high power (*p*s = .272 and .155, respectively) and low power (*p*s = .152 and .251, respectively). Nonetheless at 600 and 700 ms, *loss*, *low power* elicited marginally larger activity than *loss*, *high power* (*p*s = .053 and .082, respectively); *win*, *high power* was in tendency associated with larger activity than *win*, *low power* at 600 ms (*p* = .093) but not at 700 ms (*p* = .188); but *break-even* had similar activity in high and low power trials (*p*s = .993 and .643, respectively). At 800 ms, a marginal significant goal conduciveness effect was obtained for low power (*p* = .086), but not for high power (*p* = .257). *Loss*, *low power* had larger activity than *loss*, *high power* (*p* = .056). In contrast, wins and break-even outcomes had each similar activity on high and low power trials (*p*s = .272 and .649, respectively).

### Discussion

The aim of Experiment 1 was to test the following two predictions: (1) power appraisal drives changes in facial muscle activity and (2) goal conduciveness appraisal triggers activity changes earlier than power appraisals do.

Power appraisal effects were predicted to occur over the frontalis, corrugator, and cheek regions. Supporting this prediction, significant power appraisal effects drove corrugator activity changes around 800 ms after feedback onset with larger activity in high than in low power trials. Thus, appraisals of high power have driven a contraction of the eyebrows (cf. [27]). Over the frontalis and cheek regions no significant power main effects were observed. The CPM predicts (cf. [15]) that adequate power appraisal requires that human control over the situation is considered to be possible. Consequently, it is to be expected that power appraisal effects on facial expression will occur and be more pronounced over the frontalis and cheek regions when control appraisal has yielded the impression that the person has a sufficient degree of control to potentially change consequences.

The timing of the goal conduciveness and power appraisal main effects occurred in the predicted sequence: goal conduciveness appraisal (~600 ms) preceded power appraisal effects (~800 ms). Both main effects were found over the corrugator region. The response patterning of goal conduciveness appraisal effects was also as predicted. Frowning (increased corrugator activity) occurred in response to goal obstructive events (losses), whereas facial muscle tone was relaxed in response to goal conducive events (wins). Corrugator activity was largely similar in response to break-even outcomes and losses, indicating that break-even outcomes were similarly evaluated as goal obstructive as losses.

Furthermore, we observed interaction effects of goal conduciveness and power appraisals over both the corrugator and cheek regions. Over the corrugator region, the interaction effect occurred at 800 and 900 ms, simultaneously with the power appraisal main effect. The interaction is characterized by amplified goal conduciveness effects when power was high in contrast to invariant goal conduciveness effects when power was low. Moreover, increased eyebrow contraction in response to losses was observed only when power was high. These results suggest that, at the beginning of the appraisal-driven process, corrugator activity represents a one-to-one mapping of each appraisal result (goal conduciveness at ~600 ms and power at ~800 ms). Subsequently, as soon as power appraisal had reached preliminary closure, goal conduciveness appraisal effects were amplified in the case of high power or diminished in the case of low power appraisal.

Over the cheek region, an interaction effect was found between 600 and 800 ms after feedback onset. Although post-hoc analyses revealed only marginally significant findings, the pattern is interesting because it indicates that in high power trials the response pattern of goal conduciveness effects is congruent with a *positive valence* effect (i.e., largest activity following wins compared with losses and break-even). In contrast, in low power trials, the response pattern of goal conduciveness effects reflects a *negative valence* effect (i.e., largest activity following losses compared with wins and break-even). Furthermore, power appraisal seems to not have affected cheek region activity changes related to break-even outcomes. The cumulative interaction pattern suggests that the cheek region might reflect the *final integrative appraisal result* of the nature and pertinence of the game events rather than a one-to-one mapping of each appraisal criterion. The most interesting finding is the reversed response pattern of goal conduciveness appraisal as a function of the results of power appraisal.

To summarize, Experiment 1 showed that goal conduciveness and power appraisals affected each the corrugator region consistent with the prediction regarding the sequence and the response pattern. Indeed goal conduciveness appraisal effects preceded the ones of power appraisal over the corrugator region. The present findings over the corrugator and cheek regions lend support to the claim that power appraisal cumulatively affects the response patterning of goal conduciveness appraisal. However, the differential cumulative nature of power appraisal modulating goal conduciveness appraisal effects might be different for the corrugator (amplifying goal conduciveness effects when power is high) and cheek regions (differentiating goal conduciveness effects as function of the results of power appraisal). The CPM predicts that power appraisal is processed after control has been appraised as sufficiently high. A control appraisal manipulation might amplify the response patterns related to power appraisal. Therefore, in a second experiment, we modified the gambling task by adding a control appraisal manipulation to investigate the two issues outlined above. (1) Can the pattern of appraisal effects on the facial musculature be replicated, and (2) will the power appraisal effects be boosted, particularly over the frontalis and cheek regions by appraisals of high situational control?

## Experiment 2

The central aim of Experiment 2 was to further investigate the processing of power appraisal and its efferent effects on facial expressions by adding a control appraisal manipulation. The findings in Experiment 1 suggested sequential effects of goal conduciveness (starting ~600 ms) and power appraisals (starting ~800 ms) for the corrugator region. While the findings for power appraisal main effects for the frontalis and cheek regions were inconclusive, we observed interesting interaction effects between goal conduciveness and power appraisals for the corrugator and cheek regions. The cumulative effects of these interactions were different for the corrugator (amplification of goal conduciveness effects in high power trials) and cheek regions (reversal of goal conduciveness effects depending on power appraisal).

In Experiment 1, the absence of power appraisal main effects over the frontalis and cheek regions might have been due to the absence of conclusive results of the *control appraisal* (cf. [12]), given that control appraisal was not manipulated. This may have caused insufficient strength of efferent input of the power appraisal to drive facial muscle activity changes. The CPM (e.g., [12]) specifically predicts that when control appraisal yields an evaluation of potentially high human control in the situation, power appraisal will assess the available resources for acting on the event in a specific fashion. If no human control is possible, power appraisal would be impossible and superfluous. Consequently, an antecedent appraisal of high control should reinforce the impact of power appraisal on facial expressions. In Experiment 2, this was achieved by manipulating the perceived degree of control (low vs. high) over the gambling outcome in different blocks. Previous work on the phenomenon of *illusion of control* has shown that changes in the frequency of action-outcome contingencies in pure chance tasks changed participants’ appraisal of the degree to which they could control the task [42–45]. In these tasks, participants who frequently perceived that their action resulted in a desired outcome reported higher degrees of perceived control and vice versa.

We expected that high control appraisal would amplify the effects of power appraisal in facial muscle activity changes. Conversely, we predicted that low control appraisal would attenuate the effects of power appraisal. Interaction effects of control and power appraisals were therefore predicted for the frontalis, corrugator, and cheek regions. The predicted effects of goal conduciveness, control, and power appraisals in facial expressions are shown in Table 1.

### Materials and Methods

#### Participants

Twenty-eight female students of the University of Geneva took part. They ranged in age from 18 to 30 years (*M* = 21.21, *SD* = 3.06). They were healthy, right handed (mean of Edinburgh Handedness Inventory = 88.93, *SD* = 12.27), and had normal or corrected-to-normal vision. They were paid 25 CHF plus the bonus money they won. The study was fully approved by the Ethics Committee of the University of Geneva.

#### Experimental paradigm and procedure

**Fig 7:**
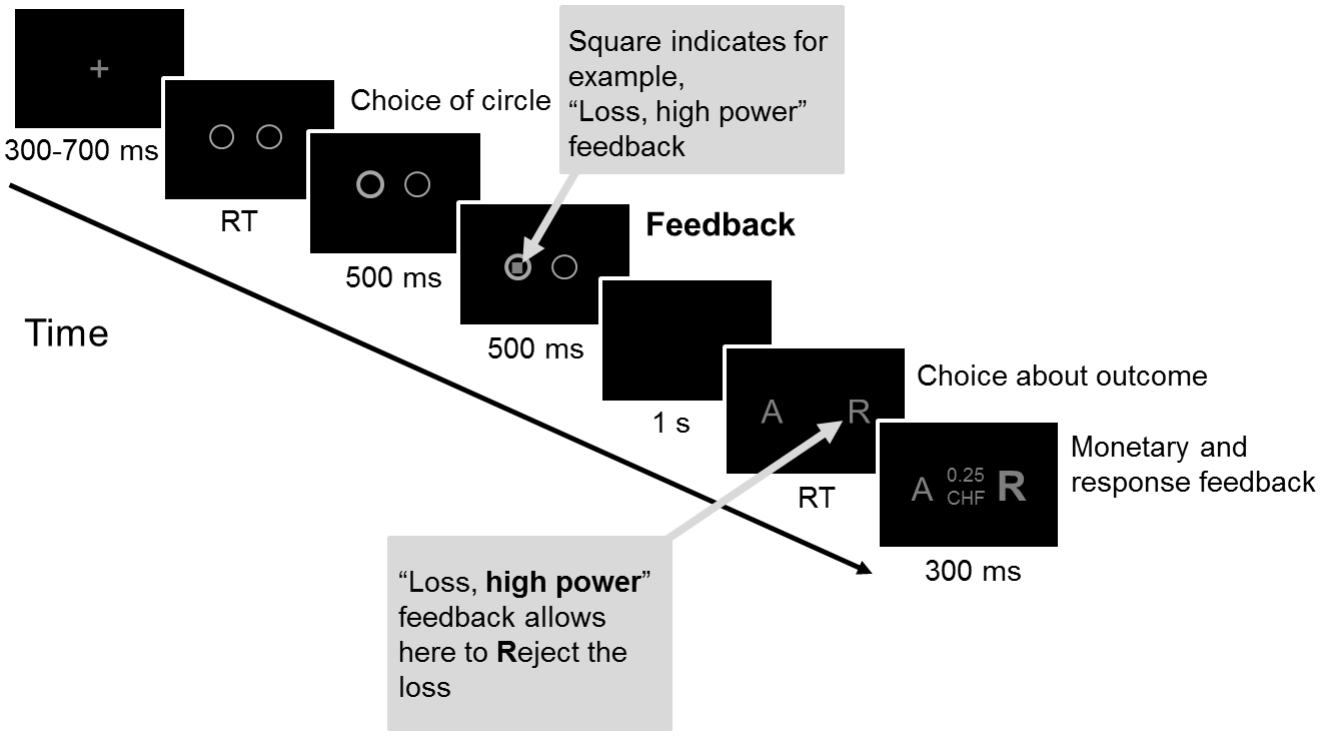
An example of a gambling task trial of Experiment 2. Presentation times of each trial event are indicated below the corresponding screen. At feedback onset, the information of goal conduciveness and power appraisals was simultaneously presented via a geometric shape in gray or black. A = accepting. R = rejecting. RT = reaction time.

The gambling task of Experiment 2 was identical to Experiment 1 (see Fig 7) except for (a) two levels of goal conduciveness (i.e., win and loss), which resulted in two presented circles (Fig 7, “Choice of circle”); (b) an increased number of trials (from 384 to 864 trials, presented in six blocks of 144 trials, randomized order) due to the control manipulation (each control block condition was presented three times); and (c) consequently, adjusted monetary magnitudes for wins and losses (+0.05 CHF and -0.05 CHF, respectively).

High control blocks provided high power feedback in 75% of the trials and low power feedback in 25% of the trials. In contrast, in low control blocks (i.e., infrequent high power feedback), the percentage of high and low power feedback was reversed. The amount of wins and losses was equal in each block (50% wins, 50% losses). Further, participants were told in the beginning of a block how much control over the upcoming trials they can expect. Thus, in the beginning of high control blocks, participants read: “In most of the trials, it will be you who decides about the outcome of a trial and you will try to find all wins,” whereas prior to a low control block, they read: “In most of the trials, it will be the computer that decides about the outcome of a trial and you will try to find all wins.” The eight feedback-stimuli conditions were as follows: (1) *loss*, *high control*, *high power* (162 trials); (2) *win*, *high control*, *high power* (162 trials); (3) *loss*, *high control*, *low power* (54 trials); (4) *win*, *high control*, *low power* (54 trials); (5) *loss*, *low control*, *high power* (54 trials); (6) *win*, *low control*, *high power* (54 trials); (7) *loss*, *low control*, *low power* (162 trials); and (8) *win*, *low control*, *low power* (162 trials).

#### Data acquisition and preprocessing

The same set-up for data acquisition, preprocessing steps, artifact rejection procedure (2.17% in total were removed), and percentage score calculation was applied as in Experiment 1.

#### Statistical analyses

Statistical analyses and report of the results are comparable to those in Experiment 1. The EMG data of each facial region were submitted to a 2 (Goal conduciveness: win vs. loss) × 2 (Control: high vs. low) × 2 (Power: high vs. low) × 9 (Time: 100-ms time-intervals) repeated measures MANOVA. In particular, goal conduciveness, control, and power were treated as within-subject variables and time as a multiple dependent variable (cf. [20]).

### Results

The results of the planned comparisons of the repeated measures MANOVAs for each facial region are summarized in Table 3. The means and standard deviations are reported in the supporting information section (S2 Table). The data of the corrugator, frontalis, and zygomaticus muscle activity changes are available in the supporting information section (S4 Data).

**Table 3:** Results of the Planned Comparisons for Each Facial Region.

		Corrugator region		Frontalis region		Cheek region	
	Time interval (ms)	*F*	ŋ²	*F*	ŋ²	*F*	ŋ²
**GC**	600	0.10	.00	2.60	.09	0.15	.01
	700	0.04	.00	4.45*	.14	0.12	.00
	800	0.89	.03	2.86	.10	0.12	.00
	900	0.16	.01	2.90	.10	0.08	.00
	1000	0.56	.02	2.17	.07	0.75	.03
	1100	0.16	.01	0.63	.02	0.26	.01
	1200	0.09	.00	1.79	.06	0.03	.00
	1300	0.22	.01	0.01	.00	0.04	.00
	1400	0.16	.01	2.60	.09	1.45	.05
**Control**	600	0.35	.01	0.51	.02	1.29	.05
	700	0.77	.03	0.78	.03	0.45	.02
	800	0.54	.02	0.47	.02	0.03	.00
	900	0.74	.03	0.16	.01	0.05	.00
	1000	2.05	.07	0.12	.00	0.16	.01
	1100	0.49	.02	1.16	.04	0.04	.00
	1200	0.77	.03	1.63	.06	0.10	.00
	1300	0.60	.02	1.70	.06	0.01	.00
	1400	0.32	.01	1.31	.05	1.03	.04
**Power**	600	1.61	.06	0.03	.00	1.70	.06
	700	2.01	.07	0.68	.02	0.95	.03
	800	2.60	.09	0.34	.01	0.63	.02
	900	0.21	.01	0.00	.00	0.80	.03
	1000	2.26	.08	4.79*	.15	0.68	.02
	1100	0.93	.03	3.35†	.11	0.19	.01
	1200	1.00	.04	0.12	.00	0.50	.02
	1300	0.09	.00	0.10	.00	2.19	.07
	1400	0.00	.00	0.02	.00	6.38*	.19
**GC × Control**	600	0.14	.01	0.02	.00	2.65	.09
	700	0.41	.01	0.44	.02	2.43	.08
	800	2.74	.09	0.51	.02	0.56	.02
	900	1.32	.05	0.05	.00	1.14	.04
	1000	1.81	.06	0.75	.03	2.02	.07
	1100	0.04	.00	0.27	.01	1.43	.05
	1200	0.06	.00	0.73	.03	2.18	.07
	1300	0.01	.00	0.36	.01	4.82*	.15
	1400	0.33	.01	1.12	.04	4.78*	.15
**GC × Power**	600	0.41	.01	0.01	.00	0.32	.01
	700	3.51†	.12	0.09	.00	0.16	.01
	800	3.61†	.12	0.47	.02	0.96	.03
	900	0.48	.02	3.61†	.12	2.20	.08
	1000	0.79	.03	0.01	.00	2.72	.09
	1100	3.07†	.10	3.02†	.10	2.43	.08
	1200	1.94	.07	2.52	.09	1.38	.05
	1300	3.08†	.10	8.24**	.23	2.43	.08
	1400	2.63	.09	0.69	.02	2.44	.08
**Control × Power**	600	1.25	.04	1.13	.04	0.11	.00
	700	2.83	.10	1.11	.04	0.11	.00
	800	1.04	.04	0.09	.00	0.15	.01
	900	1.06	.04	0.22	.01	1.54	.05
	1000	0.35	.01	0.00	.00	1.73	.06
	1100	0.95	.03	0.28	.01	0.05	.00
	1200	1.15	.04	1.75	.06	0.18	.01
	1300	3.10†	.10	0.03	.00	0.15	.01
	1400	1.13	.04	0.52	.02	0.87	.03
**GC × Control × Power**	600	2.00	.07	1.03	.04	0.11	.00
	700	0.00	.00	2.78	.09	0.35	.01
	800	0.13	.00	1.33	.05	0.91	.03
	900	0.02	.00	0.06	.00	1.46	.05
	1000	0.31	.01	0.08	.00	1.43	.05
	1100	0.74	.03	1.44	.05	2.98†	.10
	1200	0.00	.00	0.42	.02	1.91	.07
	1300	0.35	.01	3.91†	.13	0.84	.03
	1400	1.19	.04	1.83	.06	0.06	.00

*N* = 28 (Experiment 2). For each muscle region, a repeated measures MANOVA was calculated with planned comparisons. The within-subject factors were Goal conduciveness (GC: win vs. loss), Control (high vs. low), and Power (high vs. low). Time was treated as a multiple dependent variable (nine 100-ms post-stimulus time intervals).

^†^ *p* < .10.

*p < .05,

**p < .01.

#### Goal conduciveness effects

**Fig 8:**
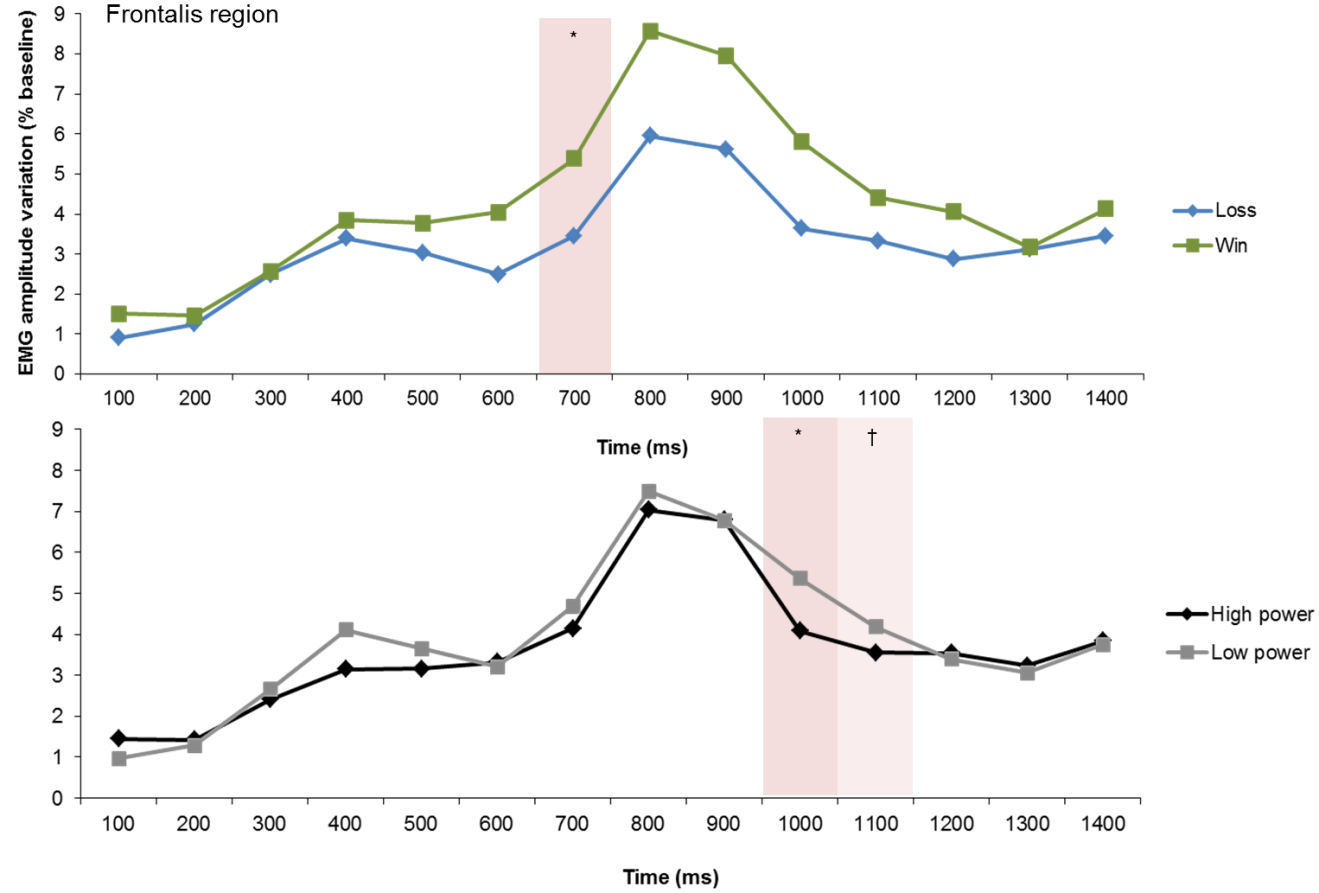
Electromyographic (EMG) amplitude variations over the frontalis region, illustrating the goal conduciveness and the power effects across time. †*p* < .10, *p < .05.

Over the frontalis region, the planned comparisons revealed a significant effect at 700 ms with larger activity in response to wins compared with losses (Fig 8). Over the corrugator and cheek regions no significant goal conduciveness effect was found.

#### Control effects

The planned comparisons did not reveal any significant main effect of control across time over the frontalis, corrugator, and cheek regions.

#### Power effects

**Fig 9:**
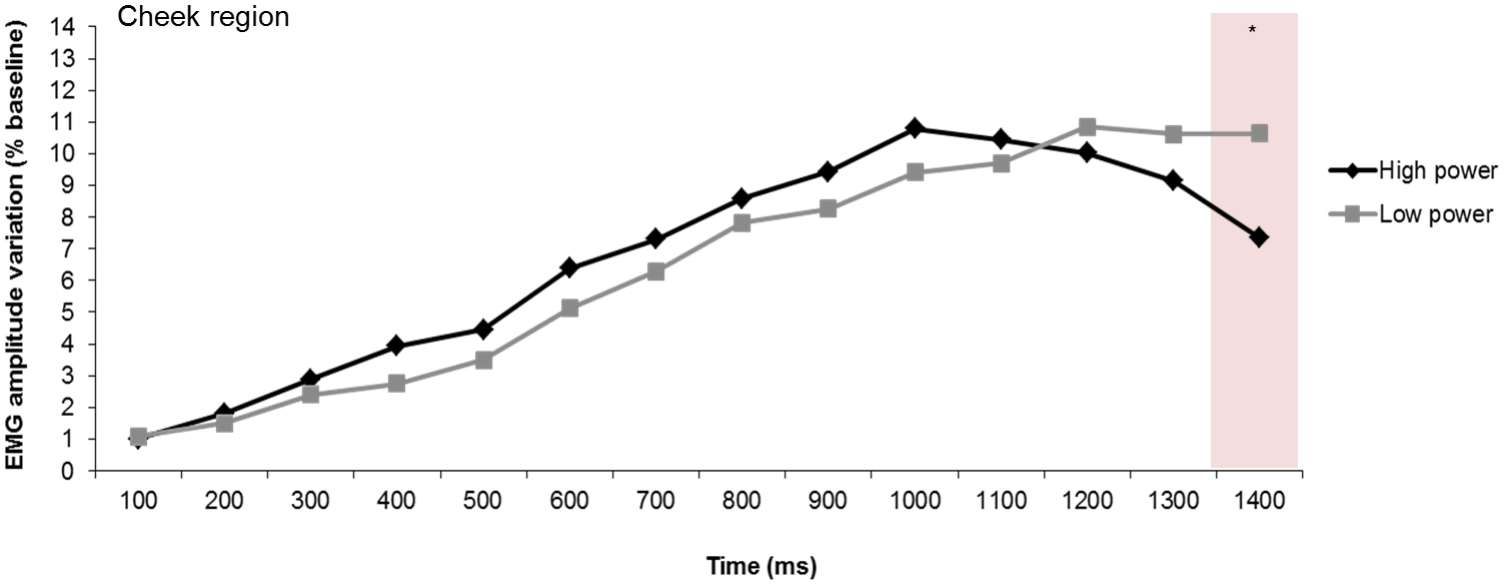
Electromyographic (EMG) amplitude variations over the cheek region, illustrating the power effects across time. *p < .05.

Over the frontalis region, the planned comparisons revealed a significant effect of power at 1000 ms and a marginally significant effect at 1100 ms, with larger activity in low power compared with high power trials (Fig 8). Over the cheek region a significant power effect was obtained at 1400 ms, showing greater activity in low power than in high power trials (Fig 9). Over the corrugator region, no significant effect of power was found.

#### Interaction effects

Two significant two-way interactions were found over the cheek (goal conduciveness by control) and frontalis regions (goal conduciveness by power). No other interaction effects were observed. Thus, the predicted interaction effect between control and power appraisals was not obtained.

#### Goal conduciveness by Control

**Fig 10:**
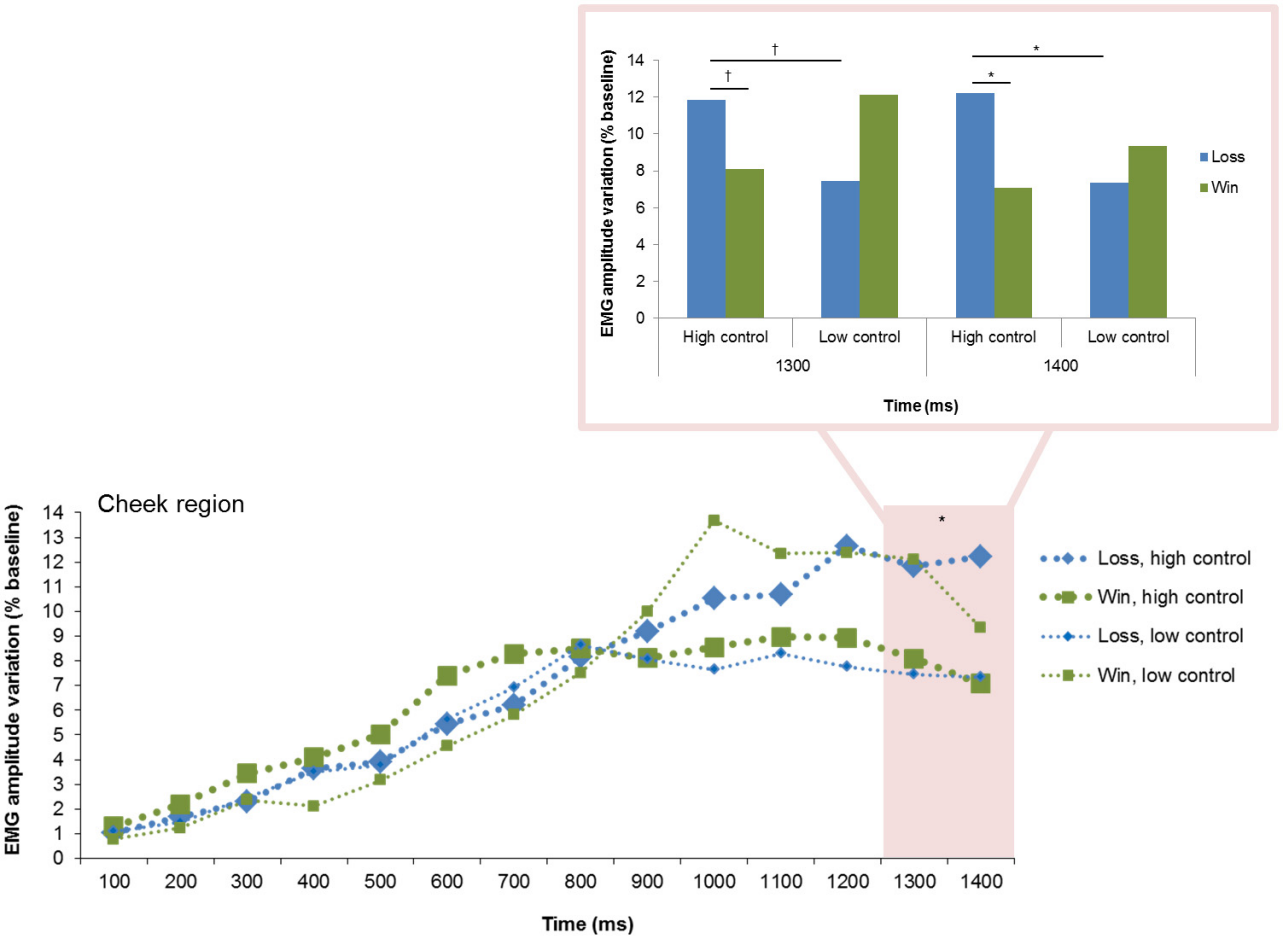
Electromyographic (EMG) amplitude variations over the cheek region, illustrating the interaction of goal conduciveness and control across time. †*p* < .10, *p < .05.

The significant two-fold interactions between goal conduciveness and control were found over the cheek region at 1300 and 1400 ms (Fig 10). At 1300 ms, post hoc contrasts revealed that *loss*, *high control* elicited marginally larger cheek activity than *loss*, *low control* (*p* = .077). In contrast, *win*, *high control* and *win*, *low control* had similar activity (*p* = .210). Further, *loss*, *high control* had marginally larger cheek activity than *win*, *high control* (*p* = .080); but *loss*, *low control* and *win*, *low control* had a similar activity (*p* = .231). At 1400 ms, post hoc tests showed that *loss*, *high control* elicited significantly larger cheek activity than *loss*, *low control*, *p* = .041, whereas *win*, *high control* and *win*, *low control* had similar activity (*p* = .234). Moreover, *loss*, *high control* had larger cheek activity than *win*, *high control* (*p* = .019); but *loss*, *low control* had similar cheek activity than *win*, *low control* (*p* = .358).

#### Goal conduciveness by Power

**Fig 11:**
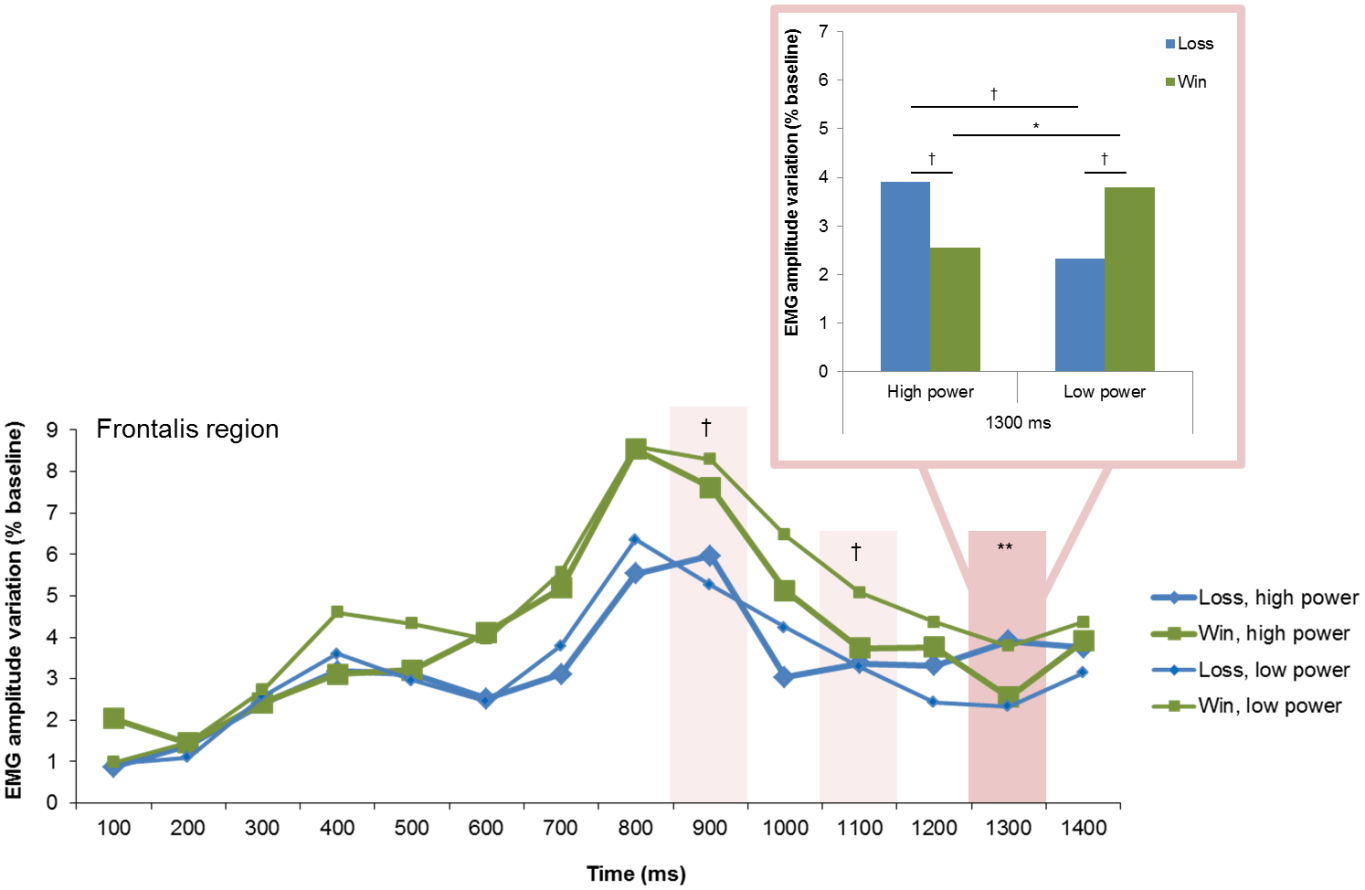
Electromyographic (EMG) amplitude variations over the frontalis region, illustrating the interaction of goal conduciveness and power across time. †*p* < .10, *p < .05, **p < .01.

A significant two-fold interaction between goal conduciveness and power was found over the frontalis region at 1300 ms, which was preceded by marginally significant interaction effects at 900 and 1100 ms (Fig 11). Post hoc contrasts at 1300 ms revealed significant greater frontalis activity following *win*, *low power* than following *win*, *high power*, *p* = .016. In tendency, *loss*, *high power* elicited greater activity than *loss*, *low power* (*p* = .092). Moreover, *loss*, *high power* had marginally larger activity than *win*, *high power* (*p* = .064) and *loss*, *low power* elicited marginally greater activity than *win*, *low power* (*p* = .057).

Over the corrugator region, only marginally significant interaction effects of goal conduciveness and power were obtained at 700, 800, 1100, and 1300 ms. In addition, a marginally significant interaction between control and power was observed at 1300 ms.

To summarize, the interaction effect of goal conduciveness and power appraisals over the frontalis region suggests in particular that high and low power appraisals affected differentially the intensity of eyebrow raising in response to wins. Low power appraisal seemed to have amplified frontalis activity in response to wins relative to high power appraisal. Nonetheless, we did not find the expected interaction effect between control and power appraisals.

### Discussion

The aims of Experiment 2 were to further investigate and to replicate the effects of power appraisal on facial EMG recordings in the presence of control appraisal. The control manipulation was introduced in the form of blocks in which perceived control was high or low. Participants were told in the beginning of each block, how much control they can expect in order to avoid different levels of expectations within and between participants.

The facial EMG recordings clearly replicated the sequential main effects of power appraisal and also of goal conduciveness appraisal. They were observed over the frontalis region and again over the cheek region, but not over the corrugator region. Over the frontalis region, first effects of goal conduciveness appraisal were found at 700 ms, which were followed by power appraisal at 1000 ms. At 1400 ms, a power appraisal main effect was additionally observed over the cheek region. Contrary to our predictions, main effects of control appraisal were inconclusive.

A possible reason for not finding control main effects might be the nature of appraisal operationalization in the gambling task. Control appraisal was manipulated across trials, participants thus evaluated the degree of control based on a series of events (i.e., action-outcome contingencies), whereas goal conduciveness and power appraisals were manipulated via geometric shapes in each trial. Consequently, the appraisal-based impact on facial expressions might be different depending on the nature of assessment (i.e., appraisal of distinct event vs. of experienced action-outcome contingencies). Different types of appraisal assessments could explain the differential effects of control appraisal relative to goal conduciveness and power appraisals. A future study which further investigates facial expressions related to control appraisal should consider an operationalization similar to that of the other appraisal criteria in the task.

With respect to the response pattern of goal conduciveness appraisal over the frontalis region, greater activity was found following goal conducive (wins) than following goal obstructive events (losses). This finding is unexpected. In a previous study [20], differential eyebrow raising was related to novelty appraisal. By assuming that eyebrow raising indicates the “positioning of sensory organs for optimal reception of stimulation” (cf. [27], p. 28), eyebrow raising in response to wins could suggest increased attention allocation towards unexpected events.

The response pattern for power appraisal revealed greater frontalis activity at 1000 ms in low power compared to high power trials confirming our predictions (see Table 1). Likewise, at 1300 ms, higher cheek activity was observed in low power relative to high power trials, which is also in line with the predictions. Low power appraisal was associated with increased eyebrow raising and cheek activity, the latter activity probably resulting from mouth stretch and lip corner retraction (cf. [12]).

Contrary to our expectation, we did not find significant interaction effects between control and power appraisals or a three-fold interaction effect of goal conduciveness, control, and power appraisals. Control appraisal effects were found only in the form of an interaction effect with goal conduciveness over the cheek region (~1300–1400 ms). Additionally, we found a power appraisal main effect at the same time and for the same facial region as this interaction effect that was characterized by larger cheek activity in response to losses relative to wins when control was high. Losses and wins elicited similar cheek activity when control was low. Moreover, *loss*, *high control* elicited greater cheek activity than *loss*, *low control*. Together, these response patterns are consistent with the general theoretical prediction that low control appraisal triggers a hypotonus of the musculature (which could result in jaw dropping or lips parting, [12]), whereas high control appraisal increases the muscular tonus. Moreover, the response pattern could reflect coping responses resulting from different *cumulated appraisal results*. For example, the final integrative appraisal result of *loss*, *high control* (i.e., event is goal incongruent and there is high control) is that goal attainment might be possible if one acted in a forceful manner. The resulting preparation of an assertive response (e.g., tight lips or mouth stretch) could be well related to amplified cheek activity. In comparison, the final appraisal result of *loss*, *low control* (i.e., event is goal incongruent and there is low control) implies that acting will not change the situation at hand and consequently, a withdrawal response could be expressed in the form of diminished cheek activity.

Although not explicitly predicted, an interaction effect between goal conduciveness and power appraisals emerged over the frontalis region. The interaction was characterized by increased eyebrow raising for losses relative to wins in high power trials, which could reflect a surprise reaction since in the event of high power the inherent expectation of obtaining a win more readily was not met (overconfidence effect, cf. [46]). In contrast, the response pattern of goal conduciveness effects was reversed in low power trials. In these trials, stronger eyebrow raising occurred in response to wins relative to losses. It is possible that in a low power context, participants were expecting a negative outcome and thus, they were surprised by a win. If eyebrow raising is an indicator of attention deployment towards unexpected events, it differed as a function of the degree of power. Thus, more attention was deployed towards wins in low power trials relative to wins in high power trials. More work on the characteristics of eyebrow raising using facial EMG is needed which extents existing knowledge about the link between appraisals and eyebrow raising.

To summarize, Experiment 2 largely replicated the temporal dynamics of sequential appraisal effects in facial expressions. In differential eyebrow raising patterns a sequential order of appraisal criteria was found evolving in complexity. First, a one-to-one mapping of goal conduciveness (~700 ms) and power appraisals (~1000 ms) dominated muscle activity changes, followed by a cumulated interaction effect of these two appraisal criteria (~1300 ms). An additional power main effect was found over the cheek region around 1400 ms, which simultaneously occurred with the interaction effect between goal conduciveness and control appraisals. This interaction effect might reflect differential coping responses based on cumulated appraisal results. The findings concerning the corrugator region were inconclusive.

## General Discussion

The two experiments reported here yielded a set of consistent results that largely support the predictions of the CPM and help to extend our knowledge about the production mechanisms for specific appraisal effects on the facial musculature as part of the component patterning in the emotion process.

The first research aim was to test the prediction that power and control appraisals directly drive facial expressions. Power appraisal effects were repeatedly found in the form of noncumulative main effects. In particular, power appraisal emerged with a response pattern largely as predicted. High power appraisal was indeed related to increased corrugator activity (contracted eyebrows ~800 ms, Experiment 1), and low power appraisal was associated with increased frontalis activity (raised eyebrows ~1000 ms, Experiment 2) and increased cheek region activity (stretched mouth and retracted mouth corners ~1400 ms, Experiment 2). In contrast, control appraisal effects were solely found in the form of an interaction effect with goal conduciveness over the cheek region (Experiment 2, ~1300–1400 ms).

In Experiment 1, we examined the feasibility of operationalizing power appraisal in a fashion that differs from the procedure in previous studies (e.g., [25, 47]). Subsequently, Experiment 2 investigated whether the power appraisal effects might be amplified when a control appraisal manipulation is added to the task. The results do not show such an effect. This may be due to the differential nature of manipulating the appraisal criteria in the task. Control appraisal was manipulated across trials, while goal conduciveness and power appraisals were directly manipulated in each trial via feedback. It might be possible that appraisal assessment based on a sequence of events drives facial expressions in a way which differs from the appraisal process in the case of concrete single events (e.g., a feedback stimulus on each trial). Future studies should consider a form of appraisal manipulation requiring similar processing of information for all appraisal criteria in the task in order to examine the expected interaction between control and power appraisals.

### Temporal Dynamics of Appraisals on Facial Expressions

The second research aim was to pursue testing of the sequence hypothesis of the CPM by extending the investigation to control and power appraisals. Consistent sequential appraisal effects confirm that appraisal-based production mechanisms indeed drive facial expressions in a fixed sequence and that power appraisal effects follow goal conduciveness effects as predicted. In the data reported here, goal conduciveness appraisal started to differentiate facial muscle activity of the upper face (Experiment 1: corrugator region; Experiment 2: frontalis region) around 600–700 ms. Subsequently, power appraisal effects were obtained over the same facial region around 800–1000 ms (Experiment 1: corrugator region; Experiment 2: frontalis region) and additionally over the cheek region around 1400 ms. The sequential effects over the frontalis and corrugator regions support the notion that initially goal conduciveness appraisal reaches preliminary closure which drove frontalis muscle activity changes. Subsequently, power appraisal produced changes in the same facial region. Finally, both appraisal criteria were integrated and produced cumulative effects on frontalis muscle activity changes.

In contrast to the findings in Experiment 1, appraisal effects occurred over the frontalis region rather than the corrugator region. It is possible that the added manipulation of control appraisal is responsible for the divergent response patterns in the upper face. However, the reasons for this divergence are difficult to determine. The added control appraisal manipulation in Experiment 2 might have changed the effects of power appraisal on facial muscle activity changes. Alternatively, adding appraisal of experienced action-outcome contingencies (i.e., control appraisal) might have involved additional cognitive processes that specifically drove frontalis muscle activity changes. It is also possible that the divergence in the results is due to individual response variability since different participants were tested in Experiments 1 and 2. Consequently, future studies need to examine whether the added control appraisal has boosted appraisal-driven frontalis muscle activity changes, whether the differential nature of cognitive appraisal processes accounts for these differential findings, or whether other variables can explain the observed pattern (e.g. interindividual variability).

The evidence is inconclusive for the sequence hypothesis with respect to control appraisal in the present experiments. It is unclear whether control appraisal is directly expressed in the face or whether it is indirectly conveyed through cumulative effects in interaction with other appraisal criteria. For example, the interaction effect of goal conduciveness and control appraisals over the cheek region suggests that control appraisal has differentially affected goal conduciveness appraisal effects around 1300–1400 ms.

To conclude, the present experiments provide an important increase in current knowledge about appraisal-based production mechanisms of facial expressions. Differential unfolding of goal conduciveness and power appraisals occurred repeatedly in a fixed sequence. As these findings confirm and extend the results of previous work as reviewed in the introduction, we can conclude that appraisal-driven effects on facial expressions unfold in the following sequence: novelty, relevance, intrinsic pleasantness, goal conduciveness, and power (and control). This holds for the very first phase of the event-specific appraisal, the sequence in the ensuing recursive appraisal processes is likely to be determined by different interacting factors.

### Cumulative Appraisal Effects

In addition to the sequence hypothesis of appraisal effects on facial expressions, the CPM predicts that each appraisal criterion of the sequence cumulatively affects the facial expressions that preceding appraisal criteria have triggered (e.g., [12, 14]). For example, a loss (goal obstruction appraisal) is expected to trigger a frown and in the next step, high power appraisal to maintain the frown and to add an expression in the lower face (e.g., lip corner retraction). In two experiments, we explored to what extent interaction effects provide evidence for *cumulative appraisal effects*. Cumulative response patterns of goal conduciveness and power appraisals were indeed found over the three facial regions. Over the frontalis region (~1300 ms), the pattern of goal conduciveness appraisal was reversed as a function of power appraisal. High power appraisal was associated with increased eyebrow raising in response to goal obstructive feedback relative to goal conducive feedback, whereas low power appraisal was related with increased eyebrow raising in response to goal conducive feedback. Over the corrugator region (~800–900 ms), high power appraisal amplified the response pattern of goal conduciveness appraisal (generally referred to as negative valence effect), while low power appraisal diminished it. Over the cheek region (~600–800 ms), the marginal interaction effect was opposite to the one found for the upper face. In particular, high power appraisal was associated with increased cheek activity in response to wins (positive valence effect), while low power appraisal was associated with increased cheek activity in response to losses (negative valence effect).

To conclude, the two experiments reported here contribute to a better understanding of cumulative appraisal effects in facial expressions. To date, only one study [22] found evidence for this CPM prediction in facial EMG recordings over the corrugator region. In the present experiments, over both the frontalis and corrugator regions, the main effects of goal conduciveness and power appraisals consistently preceded their cumulative effects. This result indicates that first a one-to-one mapping of the appraisal criteria occurred (in the predicted sequence), and subsequently cumulative effects of the two types of appraisal emerged. Moreover, in Experiment 1, cumulative effects of goal conduciveness and power appraisals were found over the cheek region. In Experiment 2, they were not replicated but a cumulative effect of goal conduciveness and control appraisals emerged over that facial region. These findings suggest that cumulative appraisal effects (i.e., interactions between appraisal criteria) express a final integrative appraisal result. Future studies should investigate whether cumulative effects are more characteristic for certain appraisal criteria rather than for others. For example, they might be typical for goal conduciveness, control, and power appraisals and rare for relevance appraisal.

### Link between Appraisals and Facial Expressions

Our results support the view that certain forms of cognitive operations (i.e., appraisals) are directly involved in driving—at least to some extent—facial expressions over the frontalis, corrugator, and cheek regions, possibly due to specific action tendencies triggered by appraisal results.

The response patterns of goal conduciveness and power appraisals over the frontalis region confirm previous research, which has related eyebrow raising to novelty appraisal [20]. Furthermore, eyebrow raising unfolded sequentially and with increasing complexity. First, each appraisal criterion triggered it differentially in the predicted sequence (goal conduciveness appraisal at 700 ms and power appraisal at 1000 ms). Subsequently, we found cumulative effects at 1300 ms. These cumulative effects indicate that under the presence of high power (i.e., agent’s action can change the outcome) eyebrow raising was observed in response to unexpected events. It suggests largely additive effects and may indicate a coping response in the form of increased attention deployment towards unexpected events when acting on the event has been appraised as possible in order to modify the consequences.

The present experiments also replicate previous findings on frowns in response to goal obstructiveness/incongruency appraisal [13, 22, 23], and enhance them by showing that high power appraisal was clearly associated with frowning. Furthermore, frowns unfolded sequentially in line with the predicted order and with increasing complexity. First, they were associated with each individual appraisal criterion (goal conduciveness appraisal at 600 ms and power appraisal at 800 ms) and subsequently with their cumulative effects (at 800 ms). In particular, the cumulative response pattern suggests that high power appraisal has enhanced frowns triggered by goal obstructiveness appraisal. The overall response pattern suggests that frowns increased (probably in both frequency and extent) in response to the motivationally most pertinent implication of the given events. Consequently, frowns communicate both a one-to-one mapping of single appraisal results (i.e., goal obstruction and high power) and an overall implication of the appraised events reflecting motivational pertinence.

Cheek region activity is in good agreement with previous work which has shown increased responses towards goal obstructive or negative events [25, 26] as well as with the prediction that low power appraisal produces mouth stretch and corner retraction. Sequential unfolding as found for the upper face was not observed in its pure form over the cheek region. Rather cumulative appraisal effects were recorded. The present experiments provide the first evidence that both control and power appraisals have an impact on cheek region activity. Low power appraisal main effects and cumulative appraisal effects (of goal conduciveness and control appraisals as well as goal conduciveness and power appraisals) suggest that the cheek region may communicate the overall implication of the event (i.e., coping potential).

The facial EMG recordings in response to gambling outcomes are consistent with the notion that the corrugator region communicates motivational pertinence of an event. In contrast, the cheek region seems to communicate the overall coping-related implication of an event. Regarding the underlying neuroanatomical network, the upper face receives more input from subcortical regions in comparison to the cheek region, which receives more cortical input [48]. Consequently, subcortical regions (e.g., brain stem, basal ganglia, limbic system) might be related to the processing of reward according to the current motivational state, whereas cortical structures (possibly predominantly the parietal cortex) are implicated in the processing of the overall implication of an event and probably also in the integration of the appraised degree of control and power, in other words, the appraised immediacy of reward. For example, the appraisal of goal conduciveness is associated with the processing of reward and can also be linked to the detection of (goal) conflict when the event makes reaching a current goal (unexpectedly) unlikely. These processes are both related to the anterior cingulate cortex [49, 50], which might be the responsible brain region for predominantly processing this type of information. Future studies need to examine the complex interrelation(s) between the anterior cingulate cortex and a resulting innervation of facial muscles.

### Limitations and Future Work

In the two experiments reported here, we investigated spontaneous emotional facial (micro-) expressions that were not posed nor elicited in a social situation. Participants were unaware of their facial expressions being recorded because they were told only that the electrodes were measuring their immediate responses toward the gambling feedback. We are still in the beginning stages of understanding the neurological basis of facial expression in relation to emotion. Future studies should take established principles of neuropsychology into account to understand what essentially drives facial expression.

Comparing the design of our two experiments and taking previous experiments that tested the production mechanisms of facial expressions into account [20, 21, 23], it seems that the number and the type of interacting appraisal criteria may differentially affect facial muscle activity. For example, without control appraisal the results over the frontalis region were inconclusive but pronounced over the corrugator region, whereas with control appraisal a clear response pattern was observed over the frontalis region but it was reduced over the corrugator region. We suggest that context (determined by the number and the type of different appraisal criteria) seems to have an important impact on facial expressions. In future studies, the impact of context on facial expression should be investigated in more detail to better understand the production mechanisms of facial response patterns. It would be beneficial to study the impact of the number and the type of manipulated appraisal criteria. Moreover, appraisal effects in facial expressions using facial EMG have not yet been studied in particular contexts such as social and/or achievement contexts.

The findings reported here could be limited in their generalizability by the fact that only right handed female students participated (due to the requirements of the simultaneously recorded electroencephalography). However, when sample size is limited it is preferable to use female participants [51]. Additionally, previous EMG results suggest that female participants show response patterns of greater magnitude which are more reliable (e.g., [51, 52]). In any case, appraisal theories do not assume gender differences in the basic functioning of cognitive processes and the resulting facial expressions.

## Conclusions

In conclusion, the two experiments showed that cognitive appraisal processes produce facial expressions. The findings support James’ [53] claim a century ago, that the overpowering “idea” of an object determines our emotional response. The present findings encourage further investigation of the production mechanisms of facial expressions. Moreover, both experiments promote the general approach to the experimental manipulation of appraisal information and the measurement of their effects over facial muscle regions using facial EMG recordings. The term *appraisal* summarizes various mechanisms involved in the immediate and automatized subjective meaning analysis of events that provides an idea about them. From this point of view, immediate facial expressions provide diagnostic cues as to what somebody thinks about an event, which seems not to be mediated by discrete emotions. With respect to research on basic emotions or classifying emotions in broad terms of valence and arousal, the present results strengthen the viewpoint that there might be only very few situations that elicit prototypical expressions linked to specific discrete emotions. Otherwise, responses to the gambling outcomes should have consistently resulted in an invariant pattern of positive expressions in response to wins (i.e., presence of smiles and absence of frowns) and negative expressions in response to losses (i.e., presence of frowns and absence of smiles).

## Supporting Information

S1 Data Data of the corrugator muscle activity recorded in Experiment 1. (XLSX)

S2 Data Data of the frontalis muscle activity recorded in Experiment 1. (XLSX)

S3 Data Data of the zygomaticus muscle activity recorded in Experiment 1. (XLSX)

S4 Data Data of the corrugator, frontalis, and zygomaticus muscle activity recorded in Experiment 2. (XLSX)

S1 Table Means (M) and Standard deviations (SD) of the Time Intervals of each Facial Region of Experiment 1. (PDF)

S2 Table Means (M) and Standard deviations (SD) of the Time Intervals of each Facial Region of Experiment 2. (PDF)

## References

[1] Fernandez-Dols JM. Advances in the study of facial expression: an introduction to the special section. Emot Rev. 2013;5: 3–7. 10.1177/1754073912457209

[2] Scherer KR, Mortillaro M, Mehu M. Understanding the mechanisms underlying the production of facial expression of emotion: a componential perspective. Emot Rev. 2013;5: 47–53. 10.1177/1754073912451504

[3] Ekman P. An argument for basic emotions. Cognition Emotion. 1992;6: 169–200. 10.1080/02699939208411068

[4] Izard CE. Innate and universal facial expressions: evidence from developmental and cross-cultural research. Psychol Bull. 1994;115: 288–299. 10.1037/0033-2909.115.2.288 8165273

[5] Galati D, Miceli R, Sini B. Judging and coding facial expression of emotions in congenitally blind children. Int J Behav Dev. 2001;25: 268–278. 10.1080/01650250042000393

[6] Galati D, Scherer KR, RicciBitti PE. Voluntary facial expression of emotion: comparing congenitally blind with normally sighted encoders. J Pers Soc Psychol. 1997;73: 1363–1379. 10.1037/0022-3514.73.6.1363 9453990

[7] Peleg G, Katzir G, Peleg O, Kamara M, Brodsky L, Hel-Or H, et al Facial expressions in various emotional states in congenitally blind and sighted subjects. Isr J Ecol Evol. 2009;55: 11–30. 10.1560/Ijee.55.1.11

[8] Russell JA. Is there universal recognition of emotion from facial expression: a review of the cross-cultural studies. Psychol Bull. 1994;115: 102–141. 10.1037/0033-2909.115.1.102 8202574

[9] Nelson NL, Russell JA. Universality revisited. Emot Rev. 2013;5: 8–15. 10.1177/1754073912457227

[10] Ellsworth PC, Scherer KR. Appraisal processes in emotion In: Davidson RJ, Scherer KR, Goldsmith HH, editors. Handbook of affective sciences. New York: Oxford University Press; 2003 p. 572–595.

[11] Roseman IJ, Smith CA. Appraisal theory: Overview, assumptions, varieties, controversies In: Scherer KR, Schorr A, Johnstone T, editors. Appraisal processes in emotion: Theory, methods, research. New York: Oxford University Press; 2001 p. 3–19.

[12] Scherer KR. The dynamic architecture of emotion: evidence for the component process model. Cognition Emotion. 2009;23: 1307–1351. 10.1080/02699930902928969

[13] Smith CA. Dimensions of appraisal and physiological response in emotion. J Pers Soc Psychol. 1989;56: 339–353. 10.1037//0022-3514.56.3.339 2926633

[14] Scherer KR. On the nature and function of emotion: a component process approach In: Scherer KR, Ekman P, editors. Approaches to emotion. Hillsdale: Lawrence Erlbaum Associates; 1984 p. 293–317.

[15] Scherer KR. Appraisal considered as a process of multilevel sequential checking In: Scherer KR, Schorr A, Johnstone T, editors. Appraisal processes in emotion: Theory, methods, research. New York: Oxford University Press; 2001 p. 92–120.

[16] Grandjean D, Sander D, Scherer KR. Conscious emotional experience emerges as a function of multilevel, appraisal-driven response synchronization. Conscious Cogn. 2008;17: 484–495. 10.1016/j.concog.2008.03.019 18448361

[17] Grandjean D, Scherer KR. Unpacking the cognitive architecture of emotion processes. Emotion. 2008;8: 341–351. 10.1037/1528-3542.8.3.341 18540750

[18] van Peer JM, Grandjean D, Scherer KR. Sequential unfolding of appraisals: EEG evidence for the interaction of novelty and pleasantness. Emotion. 2014;14: 51–63. 10.1037/A0034566 24219392

[19] Gentsch K, Grandjean D, Scherer KR. Temporal dynamics of event-related potentials related to goal conduciveness and power appraisals. Psychophysiology. 2013;50: 1010–1022. 10.1111/psyp.12079 23829421

[20] Delplanque S, Grandjean D, Chrea C, Coppin G, Aymard L, Cayeux I, et al Sequential unfolding of novelty and pleasantness appraisals of odors: evidence from facial electromyography and autonomic reactions. Emotion. 2009;9: 316–328. 10.1037/a0015369 19485609

[21] Aue T, Flykt A, Scherer KR. First evidence for differential and sequential efferent effects of stimulus relevance and goal conduciveness appraisal. Biol Psychol. 2007;74: 347–357. 10.1016/j.biopsycho.2006.09.001 17052833

[22] Aue T, Scherer KR. Appraisal-driven somatovisceral response patterning: Effects of intrinsic pleasantness and goal conduciveness. Biol Psychol. 2008;79: 158–164. 10.1016/j.biopsycho.2008.04.004 18495321

[23] Lanctot N, Hess U. The timing of appraisals. Emotion. 2007;7: 207–212. 10.1037/1528-3542.7.1.207 17352576

[24] Susskind JM, Lee DH, Cusi A, Feiman R, Grabski W, Anderson AK. Expressing fear enhances sensory acquisition. Nat Neurosci. 2008;11: 843–850. 10.1038/nn.2138 18552843

[25] van Reekum CM. Levels of processing in appraisal: evidence from computer game generated emotions Doctoral disseration, University of Geneva 2000.

[26] Lang PJ, Greenwald MK, Bradley MM, Hamm AO. Looking at pictures: affective, facial, visceral, and behavioral reactions. Psychophysiology. 1993;30: 261–273. 10.1111/j.1469-8986.1993.tb03352.x 8497555

[27] Scherer KR. Toward a dynamic theory of emotion: the component process model of affective states. Geneva Studies in Emotion and Communication. 1987;1: 1–98. Available: http://www.affective-sciences.org/system/files/biblio/1987_Scherer_Genstudies.pdf.

[28] Darwin C. The expression of the emotions in man and animals. New York: Oxford University Press; 1998.

[29] Schutzwohl A, Reisenzein R. Facial expressions in response to a highly surprising event exceeding the field of vision: a test of Darwin's theory of surprise. Evol Hum Behav. 2012;33: 657–664. 10.1016/j.evolhumbehav.2012.04.003

[30] Reisenzein R, Boerdgen S, Holtbernd T, Matz D. Evidence for strong dissociation between emotion and facial displays: The case of surprise. J Pers Soc Psychol. 2006;91: 295–315. 10.1037/0022-3514.91.2.295 16881766

[31] Grammer K, Schiefenhovel W, Schleidt M, Lorenz B, Eibleibesfeldt I. Patterns on the face: the eyebrow flash in crosscultural comparison. Ethology. 1988;77(4): 279–299. Available: http://evolution.anthro.univie.ac.at/institutes/urbanethology/resources/articles/articles/publications/flash.pdf.

[32] Smith CA, Ellsworth PC. Patterns of cognitive appraisal in emotion. J Pers Soc Psychol. 1985;48: 813–838. 10.1037//0022-3514.48.4.813 3886875

[33] Lazarus RS. Progress on a cognitive motivational relational theory of emotion. Am Psychol. 1991;46: 819–834. 10.1037//0003-066X.46.8.819 1928936

[34] Roseman IJ. Cognitive determinants of emotion: A structural theory In: Shaver P, editor. Review of personality and social psychology. 5 Beverly Hills, CA: Sage; 1984 p. 11–36.

[35] Ellsworth PC. Some implications of cognitive appraisal theories of emotion In: Strongman KT, editor. International review of studies on emotion. 1 Chichester: John Wiley & Sons Ltd; 1991 p. 143–161.

[36] Moors A. On the causal role of appraisal in emotion. Emot Rev. 2013;5: 132–140. 10.1177/1754073912463601

[37] Ekman P, Friesen WV, Hager JC. Facial action coding system: The manual: Research Nexus devision of Network Information Research Corporation, Salt Lake City, UT; 2002.

[38] Scherer KR, Zentner MR, Stern D. Beyond surprise: The puzzle of infants' expressive reactions to expectancy violation. Emotion. 2004;4: 389–402. 10.1037/1528-3542.4.4.389 15571437

[39] Pope LK, Smith CA. On the distinct meanings of smiles and frowns. Cognition Emotion. 1994;8: 65–72. 10.1080/02699939408408929

[40] Scherer KR, Ellgring H. Are facial expressions of emotion produced by categorical affect programs or dynamically driven by appraisal? Emotion. 2007;7: 113–130. 10.1037/1528-3542.7.1.113 17352568

[41] Fridlund AJ, Cacioppo JT. Guidelines for human electromyographic research. Psychophysiology. 1986;23: 567–589. 10.1111/j.1469-8986.1986.tb00676.x 3809364

[42] Langer EJ. Illusion of control. J Pers Soc Psychol. 1975;32: 311–328. 10.1037//0022-3514.32.2.311

[43] Langer EJ, Roth J. Heads I win, tails its chance: illusion of control as a function of sequence of outcomes in a purely chance task. J Pers Soc Psychol. 1975;32: 951–955. 10.1037/0022-3514.32.6.951

[44] Biner PM, Huffman ML, Curran MA, Long KR. Illusory control as a function of motivation for a specific outcome in a chance-based situation. Motiv Emotion. 1998;22: 277–291. 10.1023/A:1021300306318

[45] Wohl MJA, Enzle ME. The deployment of personal luck: sympathetic magic and illusory control in games of pure chance. Pers Soc Psychol B. 2002;28: 1388–1397. 10.1177/014616702236870

[46] Johnson DDP, Fowler JH. The evolution of overconfidence. Nature. 2011;477: 317–320. 10.1038/nature10384 21921915

[47] Kappas A, Pecchinenda A. Don't wait for the monsters to get you: A video game task to manipulate appraisals in real time. Cognition Emotion. 1999;13: 119–124. 10.1080/026999399379401

[48] Rinn WE. The neuropsychology of facial expression: a review of the neurological and psychological mechanisms for producing facial expressions. Psychol Bull. 1984;95: 52–77. 10.1037/0033-2909.95.1.52 6242437

[49] Nieuwenhuis S, Holroyd CB, Mol N, Coles MGH. Reinforcement-related brain potentials from medial frontal cortex: origins and functional significance. Neurosci Biobehav R. 2004;28: 441–448. 10.1016/j.neubiorev.2004.05.003 15289008

[50] Baker TE, Holroyd CB. Dissociated roles of the anterior cingulate cortex in reward and conflict processing as revealed by the feedback error-related negativity and N200. Biol Psychol. 2011;87: 25–34. 10.1016/j.biopsycho.2011.01.010 21295109

[51] Schwartz GE, Brown SL, Ahern GL. Facial muscle patterning and subjective experience during affective imagery: sex differences. Psychophysiology. 1980;17: 75–82. 10.1111/j.1469-8986.1980.tb02463.x 7355191

[52] Dimberg U. Facial electromyographic reactions and autonomic activity to auditory-stimuli. Biol Psychol. 1990;31: 137–147. 10.1016/0301-0511(90)90013-M 2103748

[53] James W. The physical basis of emotion (reprinted from Psychol Rev., 1984;1: 516–528). Psychol Rev. 1994;101: 205–210. 10.1037//0033-295x.101.2.205 8022955

