# Supplementary material for: Appraisals Generate Specific Configurations of Facial Muscle Movements in a Gambling Task: Evidence for the Component Process Model of Emotion
Source: PLoS One. 2015 Aug 21;10(8):e0135837. doi: 10.1371/journal.pone.0135837 (PMC4546426; doi:10.1371/journal.pone.0135837)
Supplement: S1 Table — (PDF) [file pone.0135837.s005.pdf]

S1 Table. Means (M) and Standard deviations (SD) of the Time Intervals of each Facial Region of Experiment 1.

		Corrugator region		Frontalis region		Cheek region	
	N	*M*	*SD*	*M*	*SD*	*M*	*SD*
**Loss, high power 100 ms**	24	-.73	2.84	-1.90	6.72	-.32	2.79
**Loss, high power 200 ms**	24	-.20	3.41	-.87	6.98	-.37	5.21
**Loss, high power 300 ms**	24	.77	3.24	-1.59	6.72	.08	5.90
**Loss, high power 400 ms**	24	.50	4.29	-.54	5.24	-1.53	6.60
**Loss, high power 500 ms**	24	-.68	3.67	-1.53	5.16	-2.07	6.56
**Loss, high power 600 ms**	24	-1.20	4.64	-1.59	5.60	-.66	6.05
**Loss, high power 700 ms**	24	-.33	4.72	1.66	7.02	.91	8.63
**Loss, high power 800 ms**	24	-.25	5.93	5.97	12.29	2.44	14.04
**Loss, high power 900 ms**	24	-.93	6.04	.46	6.57	2.07	13.83
**Loss, high power 1000 ms**	24	-1.36	5.42	-2.19	7.14	2.64	15.06
**Loss, high power 1100 ms**	24	.02	6.06	-1.72	6.91	2.57	12.47
**Loss, high power 1200 ms**	24	-.47	7.12	-1.14	6.40	2.92	12.81
**Loss, high power 1300 ms**	24	.41	6.23	-.62	5.01	1.50	10.77
**Loss, high power 1400 ms**	24	.16	6.47	-.95	5.15	.76	10.06
**Break-even, high power 100 ms**	24	-.20	3.01	-1.53	5.78	.65	5.98
**Break-even, high power 200 ms**	24	.99	4.25	-.44	7.34	.36	9.18
**Break-even, high power 300 ms**	24	1.77	3.96	-1.04	6.45	-1.34	7.00
**Break-even, high power 400 ms**	24	1.98	6.49	2.42	6.74	-1.38	4.52
**Break-even, high power 500 ms**	24	-1.44	5.33	1.65	5.89	-.55	6.60
**Break-even, high power 600 ms**	24	-2.08	6.25	.47	5.85	1.07	7.06
**Break-even, high power 700 ms**	24	-.32	5.40	1.56	5.40	2.75	10.23
**Break-even, high power 800 ms**	24	-1.70	5.51	4.94	8.76	3.61	10.49
**Break-even, high power 900 ms**	24	-2.50	5.78	.96	5.23	2.27	9.14
**Break-even, high power 1000 ms**	24	-2.06	5.29	-.88	5.41	2.26	7.15
**Break-even, high power 1100 ms**	24	-1.67	4.83	.01	6.85	2.26	7.73
**Break-even, high power 1200 ms**	24	-.91	4.74	-.86	6.08	1.91	8.46
**Break-even, high power 1300 ms**	24	-.56	4.46	-.56	5.49	2.96	9.49
**Break-even, high power 1400 ms**	24	.40	4.14	-.15	6.33	1.16	11.79

		Corrugator region		Frontalis region		Cheek region	
	N	*M*	*SD*	*M*	*SD*	*M*	*SD*
**Win, high power 100 ms**	24	-1.19	3.08	-2.06	5.63	.17	5.94
**Win, high power 200 ms**	24	.21	3.48	-1.00	6.84	2.10	6.49
**Win, high power 300 ms**	24	.22	3.33	-1.34	6.69	3.83	10.81
**Win, high power 400 ms**	24	-.87	3.53	-.14	4.54	4.01	10.67
**Win, high power 500 ms**	24	-2.61	5.33	-1.40	6.15	4.54	14.45
**Win, high power 600 ms**	24	-3.81	6.44	-1.62	4.84	4.97	14.81
**Win, high power 700 ms**	24	-3.61	6.17	-.25	5.27	8.80	19.65
**Win, high power 800 ms**	24	-4.74	6.97	3.12	11.68	8.31	15.38
**Win, high power 900 ms**	24	-5.18	7.41	.00	6.98	7.20	15.09
**Win, high power 1000 ms**	24	-5.27	8.09	-1.30	4.47	5.33	13.78
**Win, high power 1100 ms**	24	-4.85	7.86	-1.90	4.96	3.98	12.27
**Win, high power 1200 ms**	24	-2.70	8.13	-1.68	5.58	3.79	13.57
**Win, high power 1300 ms**	24	-3.74	7.06	-1.45	6.16	2.81	10.60
**Win, high power 1400 ms**	24	-3.00	7.09	-2.99	5.70	1.66	8.58
**Loss, low power 100 ms**	24	-.20	3.58	-1.31	6.12	-.86	4.10
**Loss, low power 200 ms**	24	-.04	3.16	-2.06	6.30	-.85	4.50
**Loss, low power 300 ms**	24	1.36	3.42	-.84	5.77	-1.26	6.03
**Loss, low power 400 ms**	24	.70	5.03	.49	5.71	-1.88	6.29
**Loss, low power 500 ms**	24	-1.87	4.34	-1.29	4.29	-.30	7.01
**Loss, low power 600 ms**	24	-3.55	4.96	-3.09	4.66	3.20	11.54
**Loss, low power 700 ms**	24	-1.71	5.49	-.39	5.79	7.35	20.89
**Loss, low power 800 ms**	24	-3.72	6.11	3.25	9.17	10.93	26.22
**Loss, low power 900 ms**	24	-4.84	6.11	-.10	6.39	10.75	28.70
**Loss, low power 1000 ms**	24	-2.82	6.66	-2.37	5.11	11.44	31.01
**Loss, low power 1100 ms**	24	-2.20	7.03	-1.75	5.38	12.26	32.36
**Loss, low power 1200 ms**	24	-2.92	6.00	-1.35	6.22	12.15	31.56
**Loss, low power 1300 ms**	24	-3.06	5.16	-1.49	6.67	8.23	26.98
**Loss, low power 1400 ms**	24	-2.03	5.20	-2.55	6.93	6.74	23.87

		Corrugator region		Frontalis region		Cheek region	
	N	*M*	*SD*	*M*	*SD*	*M*	*SD*
**Break-even, low power 100 ms**	24	-.16	3.29	-1.22	6.77	-.56	4.00
**Break-even, low power 200 ms**	24	-.02	3.62	-.44	8.37	-.65	4.07
**Break-even, low power 300 ms**	24	.29	3.47	-1.51	7.66	-.19	4.73
**Break-even, low power 400 ms**	24	1.10	5.95	.17	5.51	.69	4.72
**Break-even, low power 500 ms**	24	-.26	4.02	.43	6.14	-1.07	6.86
**Break-even, low power 600 ms**	24	-2.25	3.92	-.71	6.50	.19	8.20
**Break-even, low power 700 ms**	24	-1.31	3.94	.30	4.95	1.55	7.80
**Break-even, low power 800 ms**	24	-2.71	4.89	1.77	8.87	.87	7.19
**Break-even, low power 900 ms**	24	-3.38	5.66	-.33	5.90	1.22	9.88
**Break-even, low power 1000 ms**	24	-2.46	4.54	-2.44	5.55	1.90	11.15
**Break-even, low power 1100 ms**	24	-2.24	4.49	-1.68	6.21	.98	9.19
**Break-even, low power 1200 ms**	24	-.89	4.17	-2.53	6.14	-1.07	7.89
**Break-even, low power 1300 ms**	24	-.72	4.23	-2.26	6.72	-1.21	7.02
**Break-even, low power 1400 ms**	24	-.70	5.13	-1.87	6.86	-1.68	7.69
**Win, low power 100 ms**	24	-.49	3.15	-1.06	6.04	-.93	5.52
**Win, low power 200 ms**	24	-.33	2.67	-2.44	5.81	-2.45	5.86
**Win, low power 300 ms**	24	.28	2.73	-1.65	6.03	-2.48	6.22
**Win, low power 400 ms**	24	.02	5.01	.86	5.60	-2.23	6.36
**Win, low power 500 ms**	24	-2.41	4.50	-1.26	4.67	-2.48	6.80
**Win, low power 600 ms**	24	-4.16	4.02	-2.09	4.48	-.53	8.41
**Win, low power 700 ms**	24	-2.33	4.97	.17	7.64	1.33	11.24
**Win, low power 800 ms**	24	-4.20	4.29	4.40	13.58	2.18	13.95
**Win, low power 900 ms**	24	-4.94	6.95	.06	6.49	2.73	13.75
**Win, low power 1000 ms**	24	-4.67	6.74	-2.56	4.97	3.03	14.72
**Win, low power 1100 ms**	24	-4.51	6.94	-1.13	5.55	1.47	12.32
**Win, low power 1200 ms**	24	-3.69	6.67	-.79	6.64	2.57	11.93
**Win, low power 1300 ms**	24	-4.46	6.55	-2.12	6.73	.75	11.60
**Win, low power 1400 ms**	24	-3.46	6.18	-1.51	6.83	1.11	11.34

		Corrugator region		Frontalis region		Cheek region	
	N	*M*	*SD*	*M*	*SD*	*M*	*SD*
**Loss 100 ms**	24	-.44	2.77	-1.61	6.04	-.59	2.40
**Loss 200 ms**	24	-.22	2.74	-1.46	6.49	-.61	3.15
**Loss 300 ms**	24	.91	2.62	-1.22	5.97	-.59	3.45
**Loss 400 ms**	24	.31	4.42	-.02	4.83	-1.71	2.89
**Loss 500 ms**	24	-1.87	3.74	-1.41	4.17	-1.18	3.53
**Loss 600 ms**	24	-3.19	4.02	-2.34	4.45	1.27	6.27
**Loss 700 ms**	24	-1.98	4.64	.63	5.70	4.13	11.96
**Loss 800 ms**	24	-2.87	5.36	4.61	10.32	6.68	17.73
**Loss 900 ms**	24	-3.82	5.01	.18	5.55	6.41	17.91
**Loss 1000 ms**	24	-2.66	4.78	-2.28	3.84	7.04	18.26
**Loss 1100 ms**	24	-1.69	4.89	-1.73	4.85	7.41	17.63
**Loss 1200 ms**	24	-2.42	5.53	-1.25	4.65	7.53	16.75
**Loss 1300 ms**	24	-1.93	4.66	-1.05	4.47	4.86	13.68
**Loss 1400 ms**	24	-1.36	4.97	-1.75	4.67	3.75	12.29
**Break-even 100 ms**	24	-.18	2.62	-1.38	6.08	.04	3.74
**Break-even 200 ms**	24	.49	3.26	-.44	7.57	-.14	5.36
**Break-even 300 ms**	24	1.03	3.01	-1.27	6.75	-.77	3.96
**Break-even 400 ms**	24	1.54	5.97	1.30	5.43	-.35	2.39
**Break-even 500 ms**	24	-.85	4.17	1.04	5.38	-.81	3.72
**Break-even 600 ms**	24	-2.17	4.28	-.12	5.59	.63	4.36
**Break-even 700 ms**	24	-.81	3.73	.93	4.78	2.15	4.76
**Break-even 800 ms**	24	-2.20	4.37	3.35	8.37	2.24	4.44
**Break-even 900 ms**	24	-2.94	5.20	.31	4.96	1.74	5.34
**Break-even 1000 ms**	24	-2.26	4.71	-1.66	4.88	2.08	5.97
**Break-even 1100 ms**	24	-1.96	4.40	-.84	5.40	1.62	4.26
**Break-even 1200 ms**	24	-.90	4.11	-1.70	5.60	.42	3.92
**Break-even 1300 ms**	24	-.64	3.08	-1.41	5.38	.88	4.17
**Break-even 1400 ms**	24	-.15	4.12	-1.01	6.20	-.26	4.61
**Win 100 ms**	24	-.84	2.61	-1.56	5.62	-.38	2.84
**Win 200 ms**	24	-.06	2.87	-1.72	6.15	-.18	3.64
**Win 300 ms**	24	.25	2.59	-1.49	6.04	.67	4.88
**Win 400 ms**	24	-.43	4.03	.36	4.04	.89	4.97
**Win 500 ms**	24	-2.51	4.52	-1.33	4.60	1.03	7.67
**Win 600 ms**	24	-3.98	4.78	-1.86	3.60	2.22	8.61
**Win 700 ms**	24	-2.97	5.07	-.04	5.73	5.07	11.51
**Win 800 ms**	24	-4.47	5.25	3.76	12.01	5.24	10.85
**Win 900 ms**	24	-5.06	6.56	.03	5.97	4.97	10.30
**Win 1000 ms**	24	-4.97	6.93	-1.93	3.97	4.18	12.21
**Win 1100 ms**	24	-4.68	6.73	-1.52	4.00	2.73	10.37
**Win 1200 ms**	24	-3.20	5.46	-1.23	5.22	3.18	10.01
**Win 1300 ms**	24	-4.10	6.00	-1.78	6.24	1.78	8.51
**Win 1400 ms**	24	-3.23	5.84	-2.25	5.98	1.38	7.54

		Corrugator region		Frontalis region		Cheek region	
	N	*M*	*SD*	*M*	*SD*	*M*	*SD*
**High power 100**	24	-.71	2.49	-1.83	5.68	.17	3.25
**High power 200**	24	.34	3.11	-.77	6.73	.70	5.86
**High power 300**	24	.92	2.44	-1.32	6.17	.85	6.42
**High power 400**	24	.54	4.34	.58	4.29	.37	5.03
**High power 500**	24	-1.58	4.16	-.42	4.77	.64	5.96
**High power 600**	24	-2.36	4.91	-.91	3.85	1.79	6.51
**High power 700**	24	-1.42	4.31	.99	4.88	4.15	8.52
**High power 800**	24	-2.23	4.84	4.68	10.07	4.79	8.38
**High power 900**	24	-2.87	5.43	.47	5.42	3.84	7.02
**High power 1000**	24	-2.89	5.32	-1.46	4.25	3.41	6.88
**High power 1100**	24	-2.17	4.94	-1.21	4.92	2.94	5.97
**High power 1200**	24	-1.36	5.17	-1.23	4.98	2.87	6.42
**High power 1300**	24	-1.30	4.74	-.88	4.62	2.42	6.49
**High power 1400**	24	-.81	4.59	-1.36	4.98	1.19	5.96
**Low power 100**	24	-.29	2.59	-1.20	5.92	-.78	2.70
**Low power 200**	24	-.13	2.56	-1.65	6.23	-1.32	3.56
**Low power 300**	24	.64	2.64	-1.33	6.02	-1.31	3.95
**Low power 400**	24	.61	4.96	.51	4.63	-1.14	2.84
**Low power 500**	24	-1.51	3.76	-.71	4.10	-1.28	3.44
**Low power 600**	24	-3.32	3.51	-1.96	4.19	.95	4.35
**Low power 700**	24	-1.78	3.60	.02	4.88	3.41	9.64
**Low power 800**	24	-3.54	4.17	3.14	9.93	4.66	12.83
**Low power 900**	24	-4.39	5.17	-.12	5.11	4.90	12.94
**Low power 1000**	24	-3.32	4.54	-2.46	3.61	5.46	12.80
**Low power 1100**	24	-2.99	5.36	-1.52	4.33	4.90	11.76
**Low power 1200**	24	-2.50	4.70	-1.56	5.17	4.55	12.08
**Low power 1300**	24	-2.75	3.93	-1.95	5.48	2.59	9.69
**Low power 1400**	24	-2.06	4.50	-1.98	5.96	2.06	8.63
