# Supplementary material for: Appraisals Generate Specific Configurations of Facial Muscle Movements in a Gambling Task: Evidence for the Component Process Model of Emotion
Source: PLoS One. 2015 Aug 21;10(8):e0135837. doi: 10.1371/journal.pone.0135837 (PMC4546426; doi:10.1371/journal.pone.0135837)
Supplement: S2 Table — (PDF) [file pone.0135837.s006.pdf]

S2 Table. Means (M) and Standard deviations (SD) of the Time Intervals of each Facial Region of Experiment 2.

		Corrugator region		Frontalis region		Cheek region	
	N	*M*	*SD*	*M*	*SD*	*M*	*SD*
**Loss, high control, high power 100 ms**	28	1.59	1.66	1.09	1.88	1.02	1.59
**Loss, high control, high power 200 ms**	28	2.59	2.64	1.25	2.77	2.04	3.27
**Loss, high control, high power 300 ms**	28	2.42	4.87	2.21	3.77	2.50	4.46
**Loss, high control, high power 400 ms**	28	2.14	9.12	3.28	6.87	5.06	7.80
**Loss, high control, high power 500 ms**	28	1.22	9.68	2.05	7.56	4.05	5.37
**Loss, high control, high power 600 ms**	28	0.83	12.99	2.20	7.83	5.23	6.20
**Loss, high control, high power 700 ms**	28	1.46	15.54	3.69	9.79	6.66	7.09
**Loss, high control, high power 800 ms**	28	-0.28	15.10	6.30	10.22	8.37	8.66
**Loss, high control, high power 900 ms**	28	1.44	18.50	5.55	11.50	8.64	9.41
**Loss, high control, high power 1000 ms**	28	1.43	13.56	3.20	9.05	9.81	11.02
**Loss, high control, high power 1100 ms**	28	0.62	10.33	1.94	7.84	10.49	13.18
**Loss, high control, high power 1200 ms**	28	0.66	7.79	1.80	6.87	11.77	13.70
**Loss, high control, high power 1300 ms**	28	1.23	7.53	2.02	7.27	10.58	12.40
**Loss, high control, high power 1400 ms**	28	1.71	6.86	2.62	7.16	9.65	10.69
**Win, high control, high power 100 ms**	28	0.83	1.98	1.48	2.83	1.77	2.70
**Win, high control, high power 200 ms**	28	1.90	1.84	1.50	4.50	2.01	2.88
**Win, high control, high power 300 ms**	28	1.52	2.93	2.30	3.17	3.39	4.78
**Win, high control, high power 400 ms**	28	0.72	6.70	2.91	6.52	4.42	6.70
**Win, high control, high power 500 ms**	28	0.03	10.99	2.41	9.23	5.63	7.00
**Win, high control, high power 600 ms**	28	-0.55	8.51	2.96	9.30	8.37	11.57
**Win, high control, high power 700 ms**	28	0.42	14.02	4.55	10.80	8.35	9.35
**Win, high control, high power 800 ms**	28	-0.64	14.46	8.39	14.53	8.51	9.08
**Win, high control, high power 900 ms**	28	0.23	14.01	6.90	14.12	7.89	7.71
**Win, high control, high power 1000 ms**	28	0.20	9.75	4.57	12.47	8.41	8.05
**Win, high control, high power 1100 ms**	28	0.17	7.35	3.53	12.16	9.64	13.20
**Win, high control, high power 1200 ms**	28	-0.25	5.69	2.91	11.51	8.35	10.17
**Win, high control, high power 1300 ms**	28	0.34	5.37	2.81	9.21	8.39	11.69
**Win, high control, high power 1400 ms**	28	1.28	4.77	3.06	10.44	8.07	10.02

		Corrugator region		Frontalis region		Cheek region	
	N	*M*	*SD*	*M*	*SD*	*M*	*SD*
**Loss, high control, low power 100 ms**	28	1.07	2.66	0.82	3.10	1.03	3.50
**Loss, high control, low power 200 ms**	28	2.11	2.61	1.35	3.44	1.37	4.47
**Loss, high control, low power 300 ms**	28	2.32	4.03	2.45	9.37	2.08	4.92
**Loss, high control, low power 400 ms**	28	1.93	10.17	3.79	8.92	2.25	4.97
**Loss, high control, low power 500 ms**	28	2.86	20.21	3.12	10.40	3.76	10.74
**Loss, high control, low power 600 ms**	28	1.78	20.49	2.44	11.93	5.65	16.07
**Loss, high control, low power 700 ms**	28	2.47	17.46	4.45	14.36	5.73	11.43
**Loss, high control, low power 800 ms**	28	0.63	20.03	6.87	14.51	7.94	13.72
**Loss, high control, low power 900 ms**	28	1.82	18.77	5.11	12.75	9.77	20.15
**Loss, high control, low power 1000 ms**	28	2.34	16.16	4.53	16.45	11.29	17.40
**Loss, high control, low power 1100 ms**	28	1.17	11.50	2.54	11.71	10.87	17.34
**Loss, high control, low power 1200 ms**	28	1.23	9.63	2.06	10.82	13.55	22.76
**Loss, high control, low power 1300 ms**	28	1.87	9.61	2.39	9.91	13.11	18.26
**Loss, high control, low power 1400 ms**	28	2.25	9.21	3.59	10.99	14.77	22.16
**Win, high control, low power 100 ms**	28	0.98	2.62	1.01	2.39	0.81	3.00
**Win, high control, low power 200 ms**	28	2.26	2.78	1.82	2.80	2.37	4.57
**Win, high control, low power 300 ms**	28	2.50	7.06	2.96	5.55	3.49	9.41
**Win, high control, low power 400 ms**	28	2.23	8.94	4.61	10.51	3.78	8.69
**Win, high control, low power 500 ms**	28	2.85	13.44	5.21	13.29	4.43	9.55
**Win, high control, low power 600 ms**	28	2.17	13.81	4.59	16.06	6.41	11.24
**Win, high control, low power 700 ms**	28	3.02	18.06	6.64	17.61	8.22	14.46
**Win, high control, low power 800 ms**	28	3.19	26.27	9.43	21.75	8.49	13.02
**Win, high control, low power 900 ms**	28	1.09	16.31	8.05	17.35	8.32	13.05
**Win, high control, low power 1000 ms**	28	2.77	20.67	5.80	18.42	8.68	14.97
**Win, high control, low power 1100 ms**	28	1.77	11.65	3.64	10.88	8.29	11.39
**Win, high control, low power 1200 ms**	28	1.70	10.11	3.91	10.89	9.52	13.26
**Win, high control, low power 1300 ms**	28	2.23	6.00	2.25	8.37	7.80	11.45
**Win, high control, low power 1400 ms**	28	2.71	6.43	2.86	8.75	6.07	8.94
**Loss, low control, high power 100 ms**	28	1.04	2.68	0.64	3.29	0.42	2.36
**Loss, low control, high power 200 ms**	28	1.81	1.89	1.56	3.02	0.95	4.05
**Loss, low control, high power 300 ms**	28	2.12	3.00	2.60	4.70	1.91	6.30
**Loss, low control, high power 400 ms**	28	1.85	5.29	3.09	5.99	3.38	10.48
**Loss, low control, high power 500 ms**	28	2.57	10.96	4.16	12.40	3.88	10.22
**Loss, low control, high power 600 ms**	28	0.48	8.71	2.84	9.39	6.33	12.20
**Loss, low control, high power 700 ms**	28	1.53	12.87	2.52	11.67	7.02	16.64
**Loss, low control, high power 800 ms**	28	0.67	18.51	4.77	14.04	8.07	14.41
**Loss, low control, high power 900 ms**	28	1.23	19.73	6.39	19.10	7.27	14.87
**Loss, low control, high power 1000 ms**	28	-0.25	9.56	2.88	14.40	6.46	14.21
**Loss, low control, high power 1100 ms**	28	2.00	13.97	4.79	20.06	5.49	12.23
**Loss, low control, high power 1200 ms**	28	1.80	11.26	4.83	17.01	5.18	13.06
**Loss, low control, high power 1300 ms**	28	3.18	12.79	5.80	19.24	4.49	11.07
**Loss, low control, high power 1400 ms**	28	4.11	17.02	4.89	18.06	3.44	8.38

		Corrugator region		Frontalis region		Cheek region	
	N	*M*	*SD*	*M*	*SD*	*M*	*SD*
**Win, low control, high power 100 ms**	28	1.34	3.46	2.60	9.93	0.86	2.91
**Win, low control, high power 200 ms**	28	1.88	2.22	1.41	5.57	2.23	5.16
**Win, low control, high power 300 ms**	28	1.90	4.39	2.53	8.37	3.72	7.05
**Win, low control, high power 400 ms**	28	1.44	6.15	3.30	10.15	2.91	6.65
**Win, low control, high power 500 ms**	28	2.07	14.02	4.02	12.00	4.25	7.88
**Win, low control, high power 600 ms**	28	0.86	15.03	5.28	18.19	5.65	13.14
**Win, low control, high power 700 ms**	28	1.23	16.19	5.83	18.77	7.23	11.94
**Win, low control, high power 800 ms**	28	-0.47	18.58	8.67	24.18	9.39	11.24
**Win, low control, high power 900 ms**	28	1.53	22.29	8.33	29.08	13.89	21.07
**Win, low control, high power 1000 ms**	28	1.06	16.78	5.68	25.63	18.51	43.05
**Win, low control, high power 1100 ms**	28	1.22	16.25	3.96	22.70	16.17	39.15
**Win, low control, high power 1200 ms**	28	1.10	11.83	4.61	18.55	14.78	34.47
**Win, low control, high power 1300 ms**	28	1.48	9.79	2.30	13.47	13.09	24.34
**Win, low control, high power 1400 ms**	28	1.62	9.62	4.75	17.48	8.20	16.96
**Loss, low control, low power 100 ms**	28	0.52	1.74	1.08	3.09	1.79	2.64
**Loss, low control, low power 200 ms**	28	1.43	2.26	0.86	2.87	2.02	3.55
**Loss, low control, low power 300 ms**	28	1.58	2.09	2.71	6.76	3.02	5.59
**Loss, low control, low power 400 ms**	28	1.12	5.09	3.42	6.30	3.66	5.97
**Loss, low control, low power 500 ms**	28	0.84	7.72	2.82	6.75	3.68	5.73
**Loss, low control, low power 600 ms**	28	0.26	8.78	2.49	7.68	4.96	6.68
**Loss, low control, low power 700 ms**	28	0.34	10.23	3.16	10.83	6.83	7.75
**Loss, low control, low power 800 ms**	28	-0.31	15.01	5.85	13.99	9.24	11.22
**Loss, low control, low power 900 ms**	28	-0.38	13.08	5.42	15.03	8.88	10.07
**Loss, low control, low power 1000 ms**	28	0.36	10.92	3.96	12.96	8.84	10.62
**Loss, low control, low power 1100 ms**	28	0.76	10.50	4.05	13.81	11.12	15.92
**Loss, low control, low power 1200 ms**	28	0.90	8.80	2.80	10.47	10.35	14.73
**Loss, low control, low power 1300 ms**	28	0.94	7.77	2.28	8.96	10.44	12.75
**Loss, low control, low power 1400 ms**	28	1.21	5.85	2.70	9.18	11.26	12.46
**Win, low control, low power 100 ms**	28	1.15	1.71	0.94	2.04	0.70	2.02
**Win, low control, low power 200 ms**	28	2.27	1.93	1.12	2.32	0.26	1.82
**Win, low control, low power 300 ms**	28	1.60	2.05	2.49	4.41	1.02	2.22
**Win, low control, low power 400 ms**	28	1.19	5.05	4.60	8.64	1.33	2.44
**Win, low control, low power 500 ms**	28	0.64	6.76	3.45	9.55	2.10	2.99
**Win, low control, low power 600 ms**	28	0.28	8.57	3.33	9.15	3.44	5.22
**Win, low control, low power 700 ms**	28	1.56	14.50	4.50	14.29	4.40	6.23
**Win, low control, low power 800 ms**	28	0.93	18.18	7.80	19.23	5.65	6.25
**Win, low control, low power 900 ms**	28	0.66	15.43	8.54	24.42	6.09	7.10
**Win, low control, low power 1000 ms**	28	1.91	15.56	7.15	24.36	8.85	14.39
**Win, low control, low power 1100 ms**	28	2.13	14.45	6.51	22.38	8.51	15.15
**Win, low control, low power 1200 ms**	28	1.59	10.06	4.81	17.85	9.96	21.08
**Win, low control, low power 1300 ms**	28	1.93	8.33	5.34	15.22	11.15	24.21
**Win, low control, low power 1400 ms**	28	2.69	8.88	5.87	16.44	10.46	22.59

		Corrugator region		Frontalis region		Cheek region	
	N	*M*	*SD*	*M*	*SD*	*M*	*SD*
**High control 100**	28	1.12	1.45	1.10	1.79	1.16	1.42
**High control 200**	28	2.21	1.92	1.48	2.71	1.95	2.21
**High control 300**	28	2.19	4.38	2.48	4.04	2.86	3.75
**High control 400**	28	1.75	8.53	3.65	7.57	3.88	4.80
**High control 500**	28	1.74	13.40	3.20	9.56	4.47	6.70
**High control 600**	28	1.06	13.74	3.05	10.74	6.42	8.71
**High control 700**	28	1.84	16.12	4.83	12.69	7.24	8.02
**High control 800**	28	0.73	18.78	7.75	14.55	8.33	8.89
**High control 900**	28	1.14	16.71	6.40	13.55	8.65	9.39
**High control 1000**	28	1.68	14.74	4.52	13.68	9.55	9.57
**High control 1100**	28	0.93	9.94	2.91	10.18	9.82	10.74
**High control 1200**	28	0.83	7.98	2.67	9.71	10.79	11.31
**High control 1300**	28	1.42	6.79	2.37	8.12	9.97	9.79
**High control 1400**	28	1.99	6.42	3.03	8.98	9.64	9.97
**Low control 100**	28	1.01	1.52	1.32	4.05	0.94	1.47
**Low control 200**	28	1.85	1.35	1.24	2.50	1.36	2.13
**Low control 300**	28	1.80	2.11	2.58	5.52	2.42	3.45
**Low control 400**	28	1.40	4.63	3.60	7.16	2.82	4.44
**Low control 500**	28	1.53	9.49	3.61	9.73	3.48	4.59
**Low control 600**	28	0.47	9.87	3.49	10.66	5.10	6.21
**Low control 700**	28	1.17	13.07	4.00	13.55	6.37	6.95
**Low control 800**	28	0.21	17.32	6.77	17.39	8.09	7.56
**Low control 900**	28	0.76	17.37	7.17	21.68	9.03	9.83
**Low control 1000**	28	0.77	12.87	4.92	19.09	10.66	16.88
**Low control 1100**	28	1.53	13.51	4.82	19.21	10.32	16.90
**Low control 1200**	28	1.35	10.00	4.26	15.73	10.07	16.17
**Low control 1300**	28	1.88	8.68	3.93	14.03	9.79	14.86
**Low control 1400**	28	2.41	9.54	4.55	14.81	8.34	11.23
**High power 100**	28	1.20	1.60	1.45	3.86	1.02	1.62
**High power 200**	28	2.05	1.35	1.43	2.97	1.81	2.65
**High power 300**	28	1.99	3.08	2.41	4.26	2.88	3.68
**High power 400**	28	1.54	6.03	3.14	6.60	3.94	5.13
**High power 500**	28	1.47	11.08	3.16	9.64	4.45	4.93
**High power 600**	28	0.41	10.81	3.32	10.70	6.40	7.50
**High power 700**	28	1.16	14.33	4.15	12.39	7.31	7.06
**High power 800**	28	-0.18	16.36	7.03	14.90	8.58	6.98
**High power 900**	28	1.11	18.36	6.79	17.86	9.42	9.16
**High power 1000**	28	0.61	11.96	4.08	15.19	10.80	14.13
**High power 1100**	28	1.00	11.63	3.55	15.15	10.45	15.42
**High power 1200**	28	0.83	8.66	3.54	13.29	10.02	13.29
**High power 1300**	28	1.56	8.07	3.23	12.08	9.14	11.46
**High power 1400**	28	2.18	9.04	3.83	12.81	7.34	8.37

		Corrugator region		Frontalis region		Cheek region	
	N	*M*	*SD*	*M*	*SD*	*M*	*SD*
**Low power 100**	28	0.93	1.18	0.96	1.81	1.08	1.35
**Low power 200**	28	2.02	1.68	1.29	2.22	1.51	1.97
**Low power 300**	28	2.00	3.10	2.65	5.10	2.40	3.47
**Low power 400**	28	1.62	6.88	4.10	7.61	2.76	3.72
**Low power 500**	28	1.80	11.65	3.65	9.38	3.49	5.70
**Low power 600**	28	1.12	12.66	3.21	10.69	5.12	7.26
**Low power 700**	28	1.85	14.85	4.69	13.59	6.30	7.37
**Low power 800**	28	1.11	19.68	7.49	16.50	7.83	8.48
**Low power 900**	28	0.80	15.72	6.78	16.93	8.26	9.00
**Low power 1000**	28	1.84	15.61	5.36	17.53	9.41	10.46
**Low power 1100**	28	1.46	11.79	4.18	14.17	9.70	10.90
**Low power 1200**	28	1.35	9.37	3.40	12.07	10.84	12.52
**Low power 1300**	28	1.74	7.54	3.06	10.04	10.63	11.93
**Low power 1400**	28	2.22	7.04	3.75	10.72	10.64	12.51
**Loss 100**	28	1.05	1.11	0.91	2.21	1.06	1.29
**Loss 200**	28	1.99	1.50	1.25	2.17	1.59	2.03
**Loss 300**	28	2.11	2.75	2.49	5.54	2.38	2.94
**Loss 400**	28	1.76	6.77	3.39	6.65	3.59	5.17
**Loss 500**	28	1.87	11.67	3.04	8.77	3.84	5.65
**Loss 600**	28	0.84	12.31	2.49	8.78	5.54	6.64
**Loss 700**	28	1.45	13.75	3.45	11.12	6.56	6.72
**Loss 800**	28	0.18	16.94	5.95	12.16	8.41	7.85
**Loss 900**	28	1.03	17.31	5.62	14.23	8.64	8.96
**Loss 1000**	28	0.97	12.32	3.64	13.00	9.10	9.11
**Loss 1100**	28	1.14	11.32	3.33	13.04	9.49	9.86
**Loss 1200**	28	1.14	8.98	2.87	11.08	10.21	10.81
**Loss 1300**	28	1.81	8.93	3.12	11.11	9.66	9.31
**Loss 1400**	28	2.32	9.11	3.45	10.98	9.78	10.05
**Win 100**	28	1.07	1.53	1.51	3.34	1.03	1.75
**Win 200**	28	2.08	1.58	1.46	2.95	1.72	2.58
**Win 300**	28	1.88	3.40	2.57	3.92	2.90	4.38
**Win 400**	28	1.39	6.21	3.85	7.52	3.11	4.12
**Win 500**	28	1.40	11.07	3.77	10.41	4.10	5.41
**Win 600**	28	0.69	11.14	4.04	12.63	5.97	8.27
**Win 700**	28	1.56	15.43	5.38	14.84	7.05	8.47
**Win 800**	28	0.75	19.08	8.57	19.28	8.01	8.09
**Win 900**	28	0.88	16.75	7.95	20.62	9.05	9.48
**Win 1000**	28	1.48	15.24	5.80	19.86	11.11	16.19
**Win 1100**	28	1.32	12.08	4.41	16.87	10.65	17.02
**Win 1200**	28	1.04	8.95	4.06	14.43	10.65	16.89
**Win 1300**	28	1.50	6.55	3.17	11.09	10.11	15.88
**Win 1400**	28	2.08	6.85	4.14	12.55	8.20	11.21
